# Artificially Intelligent Tactile Ferroelectric Skin

**DOI:** 10.1002/advs.202001662

**Published:** 2020-09-03

**Authors:** Kyuho Lee, Seonghoon Jang, Kang Lib Kim, Min Koo, Chanho Park, Seokyeong Lee, Junseok Lee, Gunuk Wang, Cheolmin Park

**Affiliations:** ^1^ Department of Materials Science and Engineering Yonsei University 50 Yonsei‐ro, Seodaemun‐gu Seoul 03722 Republic of Korea; ^2^ KU‐KIST Graduate School of Converging Science and Technology Korea University 145, Anam‐ro, Seongbuk‐gu Seoul 02841 Republic of Korea

**Keywords:** artificial tactile learning electronic‐skin, ferroelectric artificial synapses, ferroelectric‐gate field‐effect transistor sensing memory, tactile sensory synapses, wearable neuromorphic electronic devices

## Abstract

Lightweight and flexible tactile learning machines can simultaneously detect, synaptically memorize, and subsequently learn from external stimuli acquired from the skin. This type of technology holds great interest due to its potential applications in emerging wearable and human‐interactive artificially intelligent neuromorphic electronics. In this study, an integrated artificially intelligent tactile learning electronic skin (e‐skin) based on arrays of ferroelectric‐gate field‐effect transistors with dome‐shape tactile top‐gates, which can simultaneously sense and learn from a variety of tactile information, is introduced. To test the e‐skin, tactile pressure is applied to a dome‐shaped top‐gate that measures ferroelectric remnant polarization in a gate insulator. This results in analog conductance modulation that is dependent upon both the number and magnitude of input pressure‐spikes, thus mimicking diverse tactile and essential synaptic functions. Specifically, the device exhibits excellent cycling stability between long‐term potentiation and depression over the course of 10 000 continuous input pulses. Additionally, it has a low variability of only 3.18%, resulting in high‐performance and robust tactile perception learning. The 4 × 4  device array is also able to recognize different handwritten patterns using 2‐dimensional spatial learning and recognition, and this is successfully demonstrated with a high degree accuracy of 99.66%, even after considering 10% noise.

## Graphical abstract

An artificially intelligent electronic skin that is capable of sensing and learning tactile stimuli is presented. This ferroelectric field effect transistor platform can implement spatial sensory synaptic functions through electrical and/or tactile spikes without complicated integration. These unique characteristics enable the demonstrated device array to recognize different handwriting patterns with a high degree of accuracy, similar to biological neural networks.

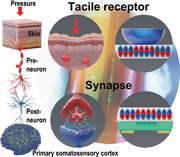

Tactile sensing artificially mimics the sensory receptors of human skin that respond to minute changes in pressure,^[^ ^1^, ^2^, ^3^, ^4^ ^]^ temperature,^[^ ^5^, ^6^, ^7^ ^]^ and humidity.^[^ ^8^, ^9^, ^10^ ^]^ This technology has attracted considerable interest due to its emerging potential use as wearable, patchable, and embedded electronic skin (e‐skin), which gives rise to numerous human‐interactive electronic materials and devices.^[^ ^11^ ^]^ Artificially intelligent e‐skin is capable of simultaneously sensing and learning from a diverse set of tactile stimuli. Thus, it is essential to develop and design an integrated sensory neural network system that can employ a nonvolatile synaptic array that is connected to tactile sensors. Considering this, a variety of integrated forms that employ synaptic devices, such as memristors^[^ ^12^, ^13^, ^14^ ^]^ and field‐effect transistor memories,^[^ ^15^, ^16^, ^17^ ^]^ combined with independent tactile sensors have been suggested and demonstrated.

However, e‐skin or sensory neural network systems, which create physical connections between sensors and memory devices, are rarely suitable for wearable and patchable devices due to their inevitably complex, interconnected, and costly fabrication steps (Table S1, Supporting Information). Additionally, these systems have a high degree of circuit resistance and require additional power‐consumption due to the number of metal wires used for system integration. This significantly deteriorates the sensitivity of tactile sensors and limits their available battery life. An e‐skin platform that is capable of concurrently learning and sensing tactile stimuli without any sensitivity degradation or physical connections has not been demonstrated thus far. Therefore, there is a need to develop a single device‐based tactile, artificial learning skin that can detect and synaptically learn about various tactile stimuli simultaneously. This type of intelligent skin would, therefore, allow for tactile stimuli to be efficiently perceived.^[^ ^18^ ^]^

In this study, we demonstrate an artificial tactile learning e‐skin platform integrated into a single device that allows for the sensing, storing, and learning of a variety of tactile information. A ferroelectric field‐effect transistor that employs pressure‐sensitive gate electrodes with nonvolatile remnant polarization was programmed with various tactile input pressures, allowing for analog conductance. Our artificial tactile learning ferroelectric skin (ATFES) has a high tactile reception sensitivity of >88 kPa^−1^, which can successfully emulate diverse tactile and essential synaptic functions, such as reliable long‐term plasticity under 10 000 input electrical spikes, as well as exhibit pressure‐spike number‐ and magnitude‐dependent plasticity. These properties allow for precise and robust tactile perception learning. Furthermore, we demonstrated that an integrated 4 × 4 ATFES array allows for 2D tactile learning and subsequent recognition of diverse handwriting patterns with an outstanding error tolerance. As a result, this method offers a novel route for designing artificially intelligent e‐skins.

Our ATFES can imitate a signal that is transmitted from a tactile sensory receptor to a primary somatosensory cortex neural network that is connected through a biological synapse between the pre‐ and postneurons, as schematically shown in **Figure** **1**a. In the ATFES, a sensory receptor is recognized by a pressure‐sensitive top‐gate electrode. The contact area with the lower ferroelectric layer can be controlled according to the magnitude of the external stimuli, as shown in Figure 1a(i),(ii). A ferroelectric dipole zone accumulates excess carriers in the semiconductor layer, which corresponds to the neurotransmitters in a biological synapse.^[^ ^19^ ^]^ This can be switched and varied according to the history of applied electrical stimuli, as well as the contact area of the gate electrode, as shown in Figure 1a(iii),(iv). The variable contact area depends on the tactile pressure, which is subsequently determined by the degree of change in channel conductance (*G*) between source (*S*) and drain (*D*) electrodes, thus allowing for changes in the drain current (*I*_DS_). In this sense, because the *G* in the ATFES is a continuously variable function that determines tactile pressure, and appropriately retains pressure‐dependent remnant ferroelectric polarization (Figure S1, Supporting Information), it can potentially mimic diverse tactile and essential synaptic functions. Consequently, these characteristics allow for a single integrated ATFES device to simultaneously learn and detect tactile inputs, which may be suitable for a variety of wearable and patchable neuromorphic applications, as shown in Figure 1a(vi).

**Figure 1:**
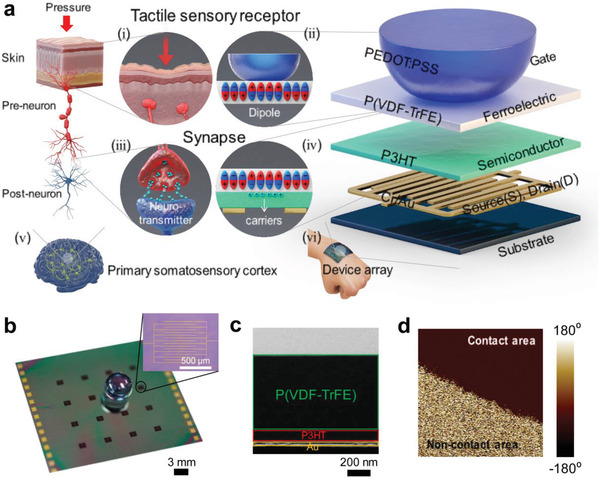
Structure of ATFES device. a) Schematic of the biological tactile perception system: (i) the tactile sensory receptor, (iii) synapse, (v) primary somatosensory cortex in brain, and the corresponding artificial tactile learning e‐skin based on Fe‐FET device: (ii) artificial tactile reception system with dome‐shaped elastomeric gate electrode and ferroelectric layer below it, (iv) the artificial synapse has excess carriers corresponding to neurotransmitters that arise from ferroelectric dipoles, (vi) an array of artificial tactile learning devices on the skin which is equivalent to the cortex neural network. b) Photograph of 4 × 4  array of ATFES device with a pressure‐sensitive PEDOT:PSS gate electrodes. The inset is an optical microscope image of Au/P3HT/P(VDF‐TrFE) of an ATFES device. c) Cross‐sectional TEM image of an ATFES device, and d) PFM image of P(VDF‐TrFE) layer at the contact area boundary.

The ATFES device has a top‐gate bottom contact field‐effect transistor (FET) structure that consists of an interdigitated Au source/drain electrode, a poly(3‐hexylthiophene‐2,5‐diyl) (P3HT) semiconductor, a poly(vinylidenefluoride‐*co*‐trifluoroethylene) [P(VDF‐TrFE)] ferroelectric polymer, and a dome‐shaped polydimethylsiloxane (PDMS) gate electrode coated with poly(3,4‐ethylenedioxythiophene)‐poly(styrenesulfonate) (PEDOT:PSS) (Figure S2, Supporting Information). PEDOT:PSS was chosen due to its pressure tolerance and its excellent mechanical flexibility.^[^ ^20^ ^]^ Although PEDOT:PSS electrode coated on the surface of a dome‐shaped PDMS was deformed upon tactile pressure, the resistance variation of an electrode was rarely observed during the deformation.^[^ ^21^ ^]^ Figure 1b depicts a photograph of the 4 × 4 ATFES array with a dome‐shaped tactile top‐gate. Source and Drain (S/D) electrodes were fabricated with the channel length and area of 40 µm and 1.2 mm^2^ on each cell, respectively, as shown in the inset of Figure 1b. Cross‐sectional transmission electron microscope (TEM) and energy dispersive X‐ray (EDX) images of the constituent atomic elements of the ATFES junction structure were taken, which clearly show discrete layers of the Au electrode, Cr adhesive layer, P3HT, and P(VDF‐TrFE), as depicted in Figure 1c; and Figure S3 (Supporting Information). When a top‐gate electrode was pressurized on the ATFES and voltage was applied between the gate and the drain electrode, a polarization switch in the ferroelectric domain occurred in the contact region, as shown in Figure 1d; and Figure S4 (Supporting Information).

**Figure** **2** shows the switching characteristics of the ATFES device, depending on the gate voltage (*V*_G_) and pressure inputs. A p‐type hysteresis transfer curve of *I*_DS_ was observed when the *V*_G_ was swept from +60 to −60 V in DC mode at a fixed pressure of 30 kPa, as shown in Figure 2a (Figures S5 and S6, Supporting Information). Two distinct ON and OFF states with ON/OFF ratio of ≈10^3^ at *V*_G_ = 0 V were observed, which occurred due to the fully saturated up and down remnant polarization domains in the P(VDF‐TrFE) layer, respectively (the inset schematics of Figure 2a). Specifically, the ATFES device exhibited an excellent ability to retain each state over a period of 20 000 s. Additionally, stable cycling endurance was observed for more than 100 times with no significant variations, as shown in Figure 2b; and Figure S7 (Supporting Information). It should be noted that the *I*_DS_ instantly switched to the ON position even when a pressure‐spike of 40 kPa for ≈50 ms was applied at a fixed *V*_G_ of −60 V (Figure S8, Supporting Information).

**Figure 2:**
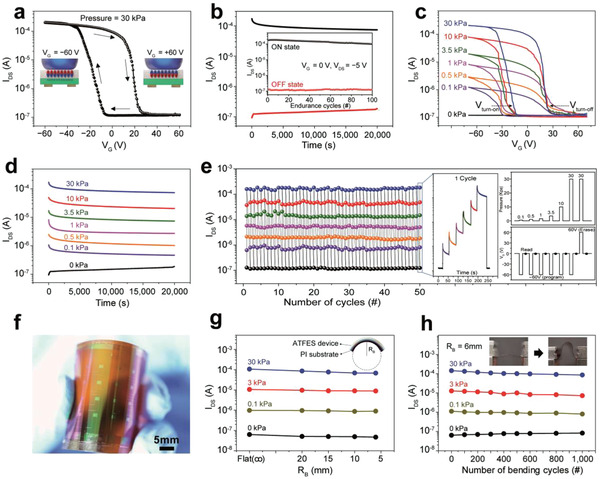
Switching and mechanical characterization of the ATFES. a) *I*_DS_ *–V*_G_ transfer curve showing current hysteresis stemmed from nonvolatile ferroelectric polarization of the P(VDF‐TrFE) layer. Pressure = 30 kPa.  b) The retention characteristic of ON and OFF states at *V*_G_ = 0 V  and *V*_DS_ = −5 V.  Inset: program/erase endurance up to 100 cycles. c) *I*_DS_ *–V*_G_ transfer curves at seven different pressures ranged from 0 to 30 kPa,  and d) the retention of each state at *V*_G_ = 0 V  and *V*_DS_ = −5 V.  The programming pressures and voltage (*V*_G_ = −60 V)  were applied for 1 s. e) Repetitive multiple program/erase switching endurance test with seven different pressures. The inset shows a real‐time response, pressures sequence, and gate voltages sequence, respectively, for one cycle. After programmed or erased, *I*_DS_ was read at *V*_G_ = 0 V  and *V*_DS_ = −5 V.  f) Photograph of a flexible ATFES array on the PI substrate, g) four distinct levels at *V*_G_ = 0 programmed by *V*_G_ = −60 V  as a function of *R*_B_, and h) bending endurance for the cycle test up to 1000 cycles under *R*_B_ = 6 mm.  The inset photograph shows a flexible ATFES array in bent and unbent states.

Because the ferroelectric polarization depends upon the contact area of the dome‐shaped gate electrode on the P(VDF‐TrFE) layer, which varies according to the pressure (Figure S9, Supporting Information), our ATFES device can detect and store various pressure levels ranging from 5 × 10^−2^ to 30 kPa. As shown in Figure 2c, the hysteresis windows for the *I*_DS_ *–V*_G_ transfer curves increased as the applied tactile pressure increased from 0 to 30 kPa, thus resulting in seven distinct states at *V*_G_ = 0 V (Figure S10, Supporting Information). Furthermore, the on and off voltages (*V*_turn‐on_ and *V*_turn‐off_) for the ON and OFF states were observed to be shifting to lower voltages.

We speculate that a reduction in the programming voltages might be associated with an increase in the effective voltage due to the reduction of the air‐gap at the interface that occurs due to an increase in pressure. In this case, higher pressure can lead to a significant change in the ferroelectric polarization at a lower electric field (Figure S11, Supporting Information). These seven states were well maintained for longer than 20 000 s without any significant degradation, as shown in Figure 2d (Figure S12, Supporting Information). It should be noted that the slight variation of *I*_DS_ at initial stage of measurement arose from the depolarization of ferroelectric layer.^[^ ^22^, ^23^ ^]^ It should be also noted that the pressure sensitivity of our ATFES is much higher at low pressure (≈88.45 kPa^−1^ at the pressure ≤10 kPa and 25.44 kPa^−1^ at the pressure >10 kPa)  (Figure S13, Supporting Information), and its minimum sensing pressure is comparable to the human skin.^[^ ^24^ ^]^ These results show the stable cycling endurance of the states programmed by different tactile pressures at *V*_G_ = ± 60 V (Figure 2e). Each multilevel tactile input cycle consisted of a sequential increase in pressure, from 0.1 to 30 kPa  with *V*_G_ = −60 V for 1 s. This was followed by a pressure of 30 kPa at *V*_G_ = +60 V for expulsion, as shown in the right inset of Figure 2e. All nondestructive switching states maintained their modified positions after 50 cycles (Figure S14, Supporting Information).

The ATFES device array was also successfully fabricated on a polyimide (PI) substrate and exhibited excellent mechanical stability during each switching state (Figure 2f–h). Multilevel hysteresis of the *I*_DS_ *–V*_G_ transfer curves was observed as a function of the tactile input pressure, similar to those on the SiO_2_ substrates (Figure S15, Supporting Information). As shown in Figure 2g,h, these multistates were driven by different pressure inputs, which were well maintained regardless of the bending radius (*R*_B_) up to 6 mm as well as the number of bending cycles (1000 times) at a fixed *R*_B_ = 6 mm (Figures S16 and S17, Supporting Information). It should be noted that the curvature strains only the active layer and channel layer since our ATFES utilizes a dome‐shaped gate electrode separated from the rest of a platform. Device responsiveness by bending itself was negligible by confirming that the *I*_DS_ was rarely varied upon the curvature without input pressure. These results, therefore, demonstrate the potential suitability of this technology for wearable and/or patchable human‐interactive electronic device platforms.

The ATFES is capable of sensing and memorizing tactile information, and can be potentially employed as a single type of neuromorphic device platform with both tactile reception and synaptic functions, as is schematically shown in **Figure** **3**a. A connected synapse is stimulated by the presynaptic spike that stems from the axon of the preneuron, which occurs due to the secretion of a neurotransmitter from the synaptic vesicle. The output signal (i.e., postsynaptic current (PSC)), so long as it exceeds a certain threshold, can then be fired at the dendrite of the postneuron.^[^ ^25^ ^]^ The generated PSC can thus be determined by the synaptic weight (*w*, synaptic strength) between the pre‐ and postneurons, which can excite or inhibit the synapse, depending on the incoming charged ions (i.e., Na^+^ and Cl^−^) through the dendrite's plasma membrane. In general, structural changes on the dendrite can be provoked through presynaptic spikes, which allows for the gradual modification of the *w*. This modification in the *w* is called “synaptic plasticity,” which has been widely recognized as the foundation for memory and learning principles in the human brain.^[^ ^26^ ^]^

**Figure 3:**
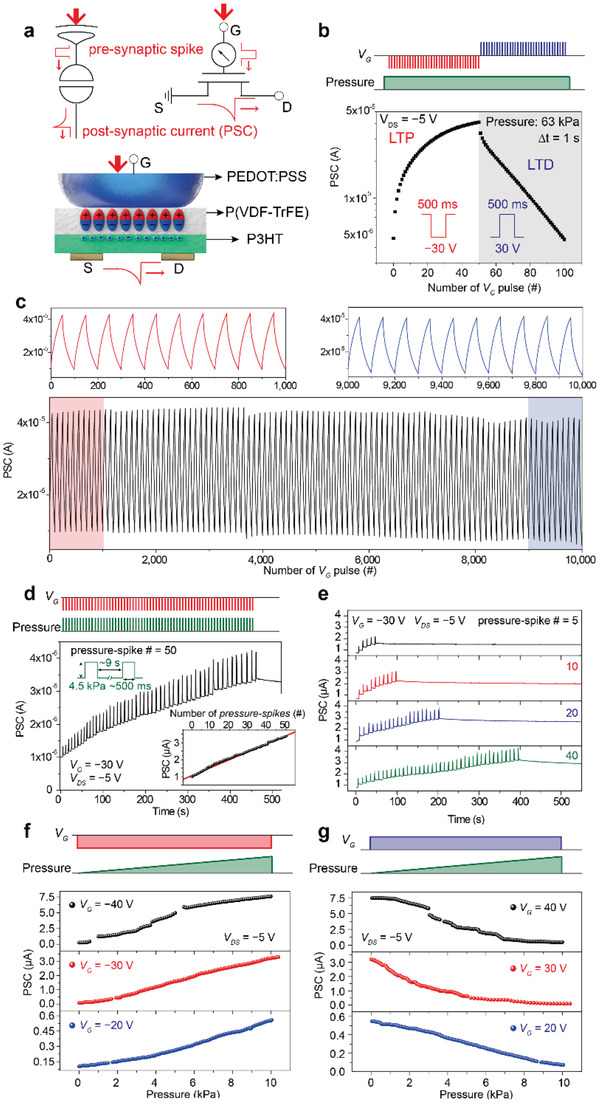
Synaptic characteristics of the ATFES. a) Schematic of the signal transmission process between pre‐ and postneurons through the ATFES. The top insets show schematics of the tactile sensory receptor/synapse and corresponding circuit diagrams for the ATFES. b) LTP and LTD of the PSC as a function of the number of *V*_G_ pulses of ±30 V  for 500 ms  at ≈63 kPa.  c) Cycling transition between the LTP and LTD for the ATFES during continuous 10 000 *V*_G_ pulses. d) Plot of the PSC response at *V*_DS_ = −5V  with respect to the 50 pressure‐spikes. Pressure = 4.5 kPa  and *V*_G_ = −30 V  for ≈500 ms.  The inset shows the PSC level as a function of the number of pressure‐spikes. e) Plots of the PSC responses with respect to the different number of pressure‐spikes (ranging from 5 to 40). The reading and programming voltages and their sequences are the same as in (d). f) LTP and g) LTD of the PSC of the ATFES as functions of the magnitude of pressure (from 5 × 10^−2^ to 10 kPa)  and the *V*_G_ (= ±20, ±30, and ±40 V) .

In the ATFES architecture (the bottom schematic of Figure 3a), the dome‐shaped top‐gate made by PEDOT:PSS plays simultaneous roles as both the tactile receptor and the axon of the preneuron. This can change the ferroelectric remnant polarization in P(VDF‐TrFE) in response to pressure and *V*_G_. The channel *G* of the P3HT semiconductor represents the *w*, determining the PSC at the postneuron flowed between the *S* and *D* electrodes. ATFES can mimic various tactile synaptic functions by adjusting the number/magnitude of pressure‐spikes, and polarity of the *V*_G_ pulses. Figure 3b shows the gradual long‐term potentiation (LTP) and depression (LTD) of the PSC at a fixed pressure (= 63 kPa), depending on the continuous potentiating and depressing input of *V*_G_ pulses (± 30 V for 500 ms) trains at a time interval (Δ*t*) of 1 s. Noticeably, excellent stability during the cycle transition (100 cycles) under the same input programming between the LTP and LTD during 10 000 continuous input pulses (Figure 3c) was also observed. It should be noted that one cycle is defined by 100 input pulses for the LTP and LTD functions. Additionally, as shown in the top graphs in Figure 3c, the first (1–1000 pulses) and last (9000–10 000 pulses) 10 cycles are almost identical, which confers to a very low cycling variability (3.18%) (Figure S18, Supporting Information). These stable LTP and LTD characteristics allow for a steady change in weight during the repeated learning process, which also makes this technology applicable as a robust tactile learning machine.

Figure 3d shows the PSC response at *V*_DS_ = −5V over time, which was triggered by a total of 50 continuous pressure‐spikes of 4.5 kPa with *V*_G_ = −30 V. The width and interval of each spike was ≈500 ms  and 9 s, respectively. The PSC level increases linearly as the number of pressure‐spikes increases and then plateaues even after the pressure‐spike is turned off, thus mimicking the tactile‐LTP function. Such PSC characteristics are common even when the number of pressure‐spikes changes from 5 to 40, as shown in Figure 3e. Because the change in PSC level appeared to be proportional to the number of pressure‐spikes, the degree of weight change could be accurately controlled (inset of Figure 3d). The linear relationship between the PSC level and the applied pressure‐spikes may, therefore, be due to a reversal in the gradual polarization in the P(VDF‐TrFE) layer with the domain wall motion along the mesh‐shaped channel interface.^[^ ^27^, ^28^, ^29^ ^]^ Figure 3f,g shows the magnitude effects of tactile pressure on LTP and LTD functions. To investigate this effect, we continuously increased the magnitude of pressure from 5 × 10^−2^ to 10 kPa through the dome‐shape PEDOT: PSS of the ATFES, while at fixed *V*_G_ = ± 20, ± 30, and ± 40 V. Because higher pressure and *V*_G_ inputs can continue to alter the direction of the ferroelectric domain and widen the domain area, the change in the PSC level will further increase at 10 kPa and *V*_G_ ± 40 V. It should be noted here that the Modified National Institute of Standards and Technology database (MNIST) pattern‐recognition accuracy was estimated to be ≈88.38%. This was based on a single neural network with a backpropagation learning algorithm that used the best fit of the LTP and LTD functions (Figure 3f,g) (Figure S19, Supporting Information).^[^ ^30^, ^31^ ^]^ This value is close to the simulated maximum accuracy in a single‐layer neural network, as previously reported.^[^ ^32^, ^33^ ^]^

Considering the aforementioned discussion, the ATFES architecture holds great potential as a single artificially intelligent tactile skin that is capable of simultaneously sensing, learning, and recognizing tactile information. To demonstrate the potential for 2D spatial tactile mapping and pattern recognition, we fabricated a 4 × 4 pixelated array based upon the ATFES device with high uniformity (Figure S20, Supporting Information). A dome‐shaped PDMS array was coated with a PEDOT:PSS, as shown in the photograph in **Figure** **4**a (Figure S21, Supporting Information). Because some ATFES devices can be programmed using a commercial touch pen, we were able to write the alphabet in the array. For example, “*N*” was encoded by utilizing the difference in the magnitude of pressure‐spike (from 5 × 10^−2^ to 40 kPa) at a fixed *V*_G_ = −30 V, as schematically shown in Figure 4b. As a result, the Δ*w*/*w*_o_ for each node in the 4 × 4 array which corresponded to “*N*” would have a different value, as shown in Figure 4c. It should be noted that *w*_o_ is defined as the initial *w* for each node and each PSC response was measured at *V*_DS_ = −5 V and *V*_G_ = 0 V. However, when different users write the “*N*” on the array using a touch pen, each “*N*” pattern will be encoded differently depending on their handwriting styles. In other words, these encoded PSC levels in the array were different according to the unique pen pressures for the “*N*” patterns. For example, as shown in Figure 4d, there are three different *N* patterns (*N*_1_, *N*_2_, and *N*_3_), encoded by three different users, which can have different PSC levels for each pixel on the array.

**Figure 4:**
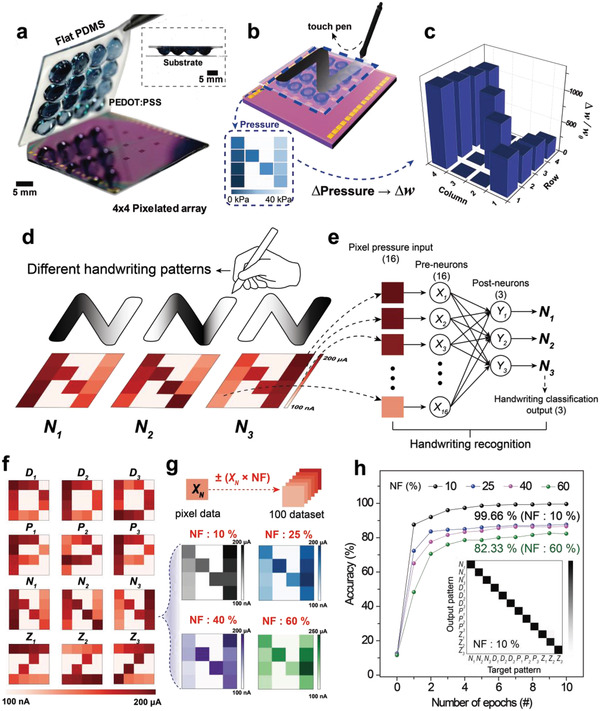
2‐dimensional spatial tactile mapping and handwriting pattern recognition of a 4 × 4 ATFES array. a) Photograph of 4 × 4 pixelated ATFES array combined with dome‐shaped PEDOT:PSS gate electrodes on the flat PDMS. The inset shows a cross‐sectional photograph of the ATFES array. b) Schematic illustration of the alphabet “*N*” pattern written by a commercial touch pen on the 4 × 4 ATFES array. Inset shows the “*N”* pattern encoded by the different magnitude of pressure‐spike (from 5×10 ^−2^ to 40kPa)  at *V_G_* = −30V.  c) The histogram of *Δw/w_o_* in the 4 × 4 ATFES array. d) Schematic illustrations of three different handwriting styles for the “*N*” patterns (*N_1_*, *N_2_*, and *N_3_*). The bottom inset shows the contour plot of corresponded PSC levels as examples. e) Constituents of a single‐layer neural network for the handwriting pattern recognition. f) The contour plots of the measured PSC levels for four alphabet characters (“*D*,” “*P*,” “*N*,” “*Z*”) encoded by three different handwriting styles (1, 2, 3). g) Examples of four “*N_3_*” pattern dataset were generated by different NF values (10%, 25%, 40%, and 60%). h) Recognition accuracy for the handwriting patterns during 10 learning epochs with different NF values. Inset shows the confusion matrices between the output and target patterns for a classification test of 12 alphabetical character sets for NF = 10%.

A single‐layer neural network with a sigmoid function was employed as a classifier for the three different handwriting patterns that were encoded on the 4 × 4 ATFES array, as shown in Figure 4e. The 16 pixels of each *N* pattern in the array were individually connected to the pre‐neurons (*X*_1_, *X*_2_, …, and *X*_16_) in order. The postneurons (*Y*_1_, *Y*_2_, and *Y*_3_) were assigned to three different handwriting patterns, such as *N*_1_, *N*_2_, and *N*_3_. The learning algorithm and fitting parameters were the same as those used in above MNIST pattern recognition simulation (Figure S19, Supporting Information). As shown in Figure 4f, four alphabetical characters (“*D*,” “*P*,” “*N*,” and “*Z*”) were experimentally encoded according to three different handwriting styles (i.e., *N*_1_, *N*_2_, and *N*_3_) in the ATFES array, for a total of 12 different classes (*D*_1_ *, D*_2_ *, D*_3_ *, P*_1_ *, P*_2_ *, P*_3_ *, N*_1_ *, N*_2_ *, N*_3_ *, Z*_1_ *, Z*_2_, and *Z*_3_).

It should be noted that the histogram of Δ*w*/*w*_o_ for the corresponding patterns exhibited a PSC response that was measured at each node in the array (Figure S22, Supporting Information). Following this, we applied a noise signal to each class by multiplying a random number within the noise factor (NF), ranging from 0.1 (10%) to 0.6 (60%). We then created 100 individual augmented data per class to train and test the datasets (top schematic in Figure 4g). Figure 4g depicts an example of the changed *N*_3_ class according to the different NF values (10%, 25%, 40%, and 60%). Additional examples are also shown in Figures S23–S26. As the NF continuously increased, the original pattern (NF = 0%) largely varied and was difficult to classify. Figure 4h shows the recognition accuracy results for the four handwritten alphabetical letters as functions of the learning epochs and NF values.

Based on this network, we achieved a surprising 99.66% accuracy at 10 epochs, even for NF = 10%. This results in an almost perfectly diagonal pattern in the confusion matrices between the target and output patterns (inset of Figure 4h). This supports the potential of the 2D tactile mapping and pattern recognition of the ATFES array, which can be utilized for security coding and personal identifying systems. Even at NF = 60%, the accuracy was still high enough (≈82.33%) to decipher the handwritten information. This indicates that a neural network that consists of ATFES devices is tolerant of tactile error‐signals. And, the details of learning results including confusion matrices are described in Figure S27 (Supporting Information).

This work demonstrated that a ferroelectric polymer‐gated organic FET can be used as a single, integrated e‐skin platform. This technology can simultaneously sense and learn a variety of tactile information in a synaptic manner, especially when combined with a dome‐shaped elastomeric tactile gate electrode. Based on our ATFES, a reliable and diverse set of essential synaptic functions were successfully demonstrated, including the stable transition of LTP/LTD during 10 000 electrical input pulses, low variability of 3.18%, and pressure‐spike number/magnitude‐dependent plasticity. Furthermore, based on the 4 × 4 ATFES array, three different handwriting styles patterns were recognized for four alphabetical letters (“D,” “P,” “N,” and “Z”). This test achieved over 99% accuracy, even at a 10% NF. Thus, considering our results, this ATFES offers a novel route for designing artificial intelligent e‐skins with a high degree of precision that are capable of error‐tolerant tactile perception learning.

## Experimental Section

##### Materials

P(VDF‐TrFE) (*M*_w_ = 400 000 g mol^−1^) with a 25% mol fraction of TrFE was purchased from Solvay. PDMS (Sylgard 184) and crosslinkers were purchased from Dow Corning. P3HT (*M*_w_ = 180 000 g mol^−1^) with 98.5% head‐to‐tail regioregularity, poly(methyl methaacrylate) (PMMA) (*M*_w_ = 120 000 g mol^−1^) was purchased from Sigma‐Aldrich, Korea. The PEDOT:PSS (Clevios PH 1000) was modified through mixing with 5 wt% DMSO and 1 wt% Zonyl surfactant (FS‐300 fluoro‐surfactant from Aldrich) with respect to PEDOT:PSS. All the organic solvents, including 2‐butanone (methyl ethyl ketone (MEK)), dimethyl sulfoxide (DMSO), and toluene were purchased from Sigma‐Aldrich, Korea.

##### Device Fabrication

An SiO_2_ substrate was cleaned in an ultrasonic bath with acetone and 2‐propanol for 1 h each, while a PI substrate that was used to make a flexible device was cleaned for 2 h. First, the Cr/Au (1 nm/30 nm thick) source/drain (S/D) electrodes were thermally evaporated on a substrate that was patterned with photolithography. Subsequently, a P3HT solution in Toluene (1 wt%) was spin‐coated on the source/drain electrodes at 2000 rpm for 60 s, followed by PMMA solution in acetone (5 wt%) at 2000 rpm for 60 s. After thermal treatment at 60 °C for 30 min  to remove any residual solvent, a 100 nm thick layer of Cu was deposited using thermal evaporation with a patterned shadow mask. The device was treated using reactive ion etching (RIE), while the Cu film served as an RIE blocking mask. After RIE etching, the device was immersed in acetone for 30 min to remove the PMMA sacrificial layer and Cu film. Then P(VDF‐TrFE) in MEK (7 wt%) was spin‐coated on patterned P3HT layer at 2000 rpm for 60 s in a ferroelectric insulator. After thermal treatment at 60 °C for 30 min to remove any residual solvent, the device was heat‐treated again at 135 °C for 2 h to enhance the properties of P3HT (mobility)^[^ ^34^ ^]^ and P(VDF‐TrFE) (crystallinity).^[^ ^35^ ^]^ In order to create the pressure‐sensitive gate electrode, the PDMS (prepolymer and curing agent ratio of 10:1) was poured onto a dome‐shaped Si mold and subsequently annealed at 80 °C for 12 h to harden it. This was followed by UV treatment for 20 min. A PEDOT:PSS film was then dip‐coated onto the UV‐treated surface on the PDMS hemisphere. The film was annealed at 100 °C for 15 min in under ambient conditions.

##### Device Characterization

Transistor properties and synaptic characteristics measurements were determined using a Keithley 4200 semiconductor characterization system and a semiconductor parameter analyzer (4155C, Keysight) equipped with a pulse generator (81104A, Keysight). Pressure was applied and measured using *z*‐axis pressure equipment combined with force gauges. The thickness of the P(VDF‐TrFE) and P3HT films was measured using a Surface Profiler (DektakXT) (Bruker Co.). The cross‐sectional view and EDX mapping were examined using a TEM (JEM‐F200). The polarization switching behavior of P(VDF‐TrFE) films was investigated using a commercial atomic force microscopy (Multimode SPM) (Bruker Co.) with piezoresponse force microscopy (PFM) mode. The polarization‐electric field hysteresis loops were obtained using a virtual ground circuit (Radiant Technologies Precision LC unit) in a dark box at room temperature (298 K).

## Conflict of Interest

The authors declare no conflict of interest.

## Supporting information

Supporting Information
