# Supplementary material for: Artificially Intelligent Tactile Ferroelectric Skin
Source: Adv Sci (Weinh). 2020 Sep 3;7(22):2001662. doi: 10.1002/advs.202001662 (PMC7675051; doi:10.1002/advs.202001662)
Supplement: Supplementary file 1 — Supporting Information [file ADVS-7-2001662-s001.pdf]

Copyright WILEY-VCH Verlag GmbH & Co. KGaA, 69469 Weinheim, Germany, 2020.

Supporting Information

Artificially Intelligent Tactile Ferroelectric Skin

Kyuho Lee, Seonghoon Jang, Kang Lib Kim, Min Koo, Chanho Park, Seokyeong Lee, Junseok Lee, Gunuk Wang and Cheolmin Park**

Device type	Components	Number of components [#]	Sensitive part	Number of states [#]	Ratio (w/ to w/o stimuli)	Endurance cycles [#]	Retention [s]	Ref.
Pressure memory	Transistor + Transistor	2	Rubber/Cu	2	~10^4	~10^3	~10^3	[1]
	Resistor + Resistor	2	Rubber/Au	2	~10^5	N/A	~10^7	[2]
	Resistor + Resistor	2	MIicro-pyramid PDMS/Ag NWs	3	~10^7	50	~10^3	[3]
	Resistor + Resistor	2	Conductive rubber	2	~10^5	40	~10^3	[4]
	Resistor + TENG	2	Al/PTFE	2	~10	80	N/A	[5]
	Resistor + Transistor	2	Conductive rubber	2	~40	600	~10^3	[6]
	Resistor + Transistor	2	Conductive paper	2	< 10	25	~10^4	[7]
	Transistor + Transistor	2	Suspended gate	Multi-state (Synaptic)	~10^2	N/A	N/A	[8]
	Resistor + Transistor	2	Micro-pyramid PDMS/CNTs	Multi-state (Synaptic)	~10^5	N/A	N/A	[9]
	Resistor + Resistor	2	Micro-pyramid PDMS/Au	Multi-state (Synaptic)	N/A	> 7000	N/A	[10]
	Resistor + Resistor + Piezoelectric sensor	3	Piezoelectric sensor device	Multi-state (Synaptic)	N/A	~10^{12}	N/A	[11]
Strain memory	Transistor + Nanogenerator	2	Al/P(VDF-TrFE)/ Au	Multi-state (6)	~10^3	~10^2	~10^3	[12]
	Resistor + Resistor	2	Gold film	2	~10^2	~10^3	~10^3	[13]
**Our ATFES device**	**1-Transistor**	**1**	**PEDOT:PSS/Dome-shaped PDMS**	**Multi-state (Synaptic)**	**~10^3**	**> 50**	**~10^4**	**This work**

Table S1. Summary of the reported tactile sensing memories. Tactile memories demonstrated with the physical combination of independent sensors and memories.

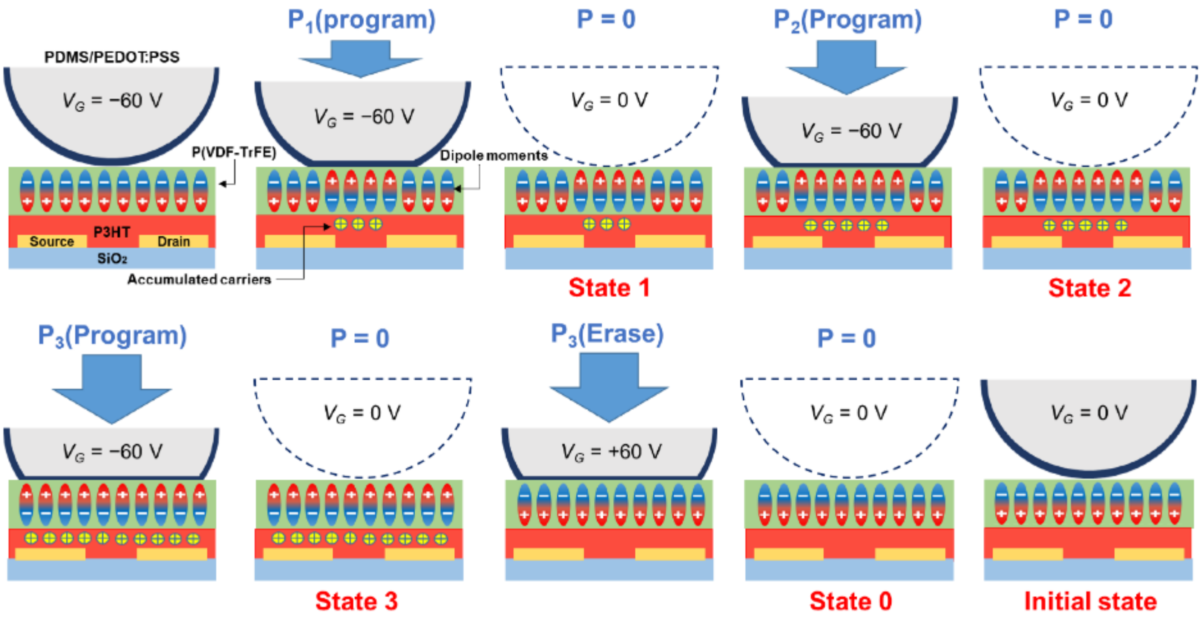

Figure S1. Schematic illustrating the multi-level tactile memory mechanism in the ATFES device. The channel conductance (*G*) can be retained due to the pressure-dependent remnant ferroelectric polarization. Pressure increased sequentially from P_1 to P_3.

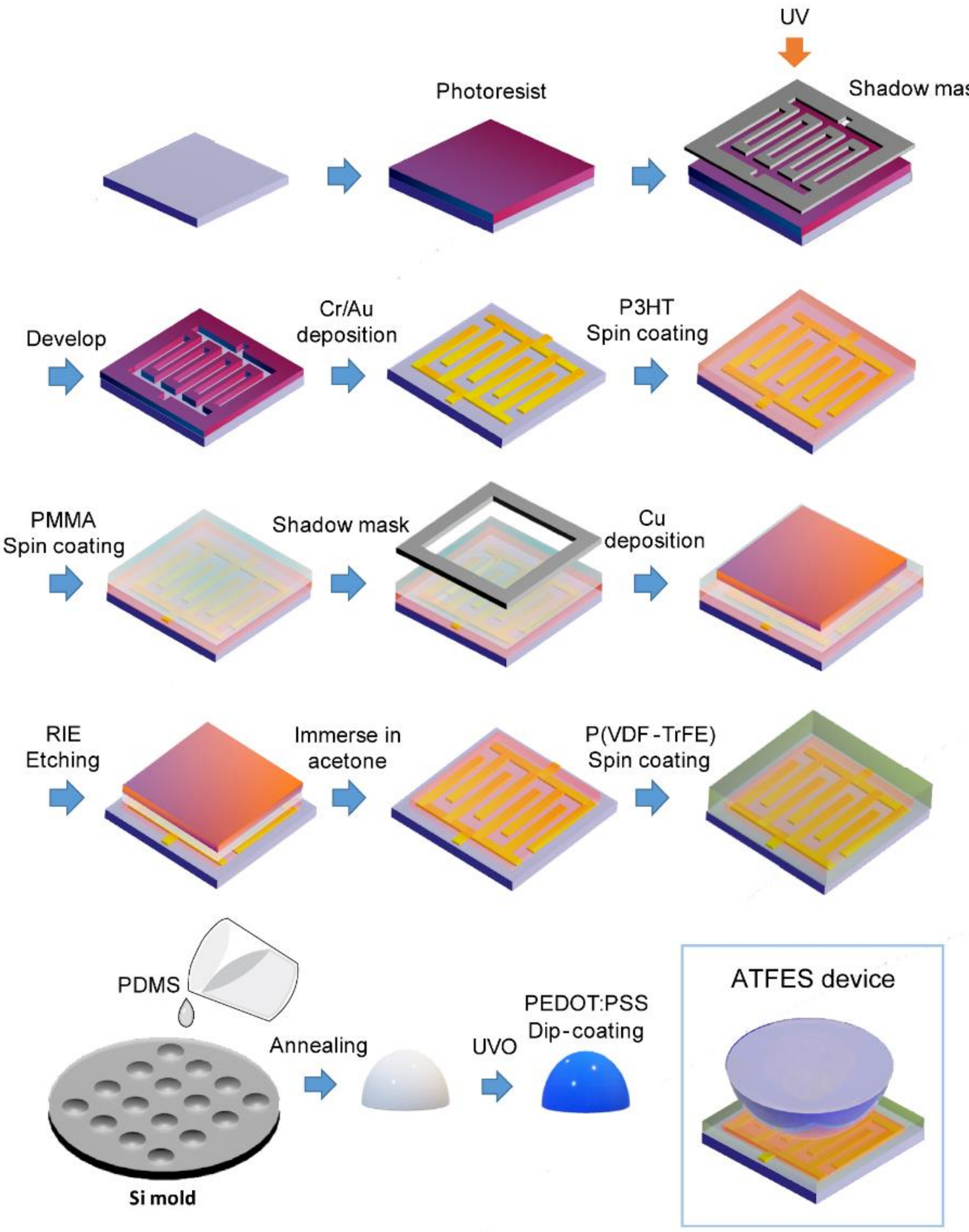

Figure S2. Device fabrication processes.

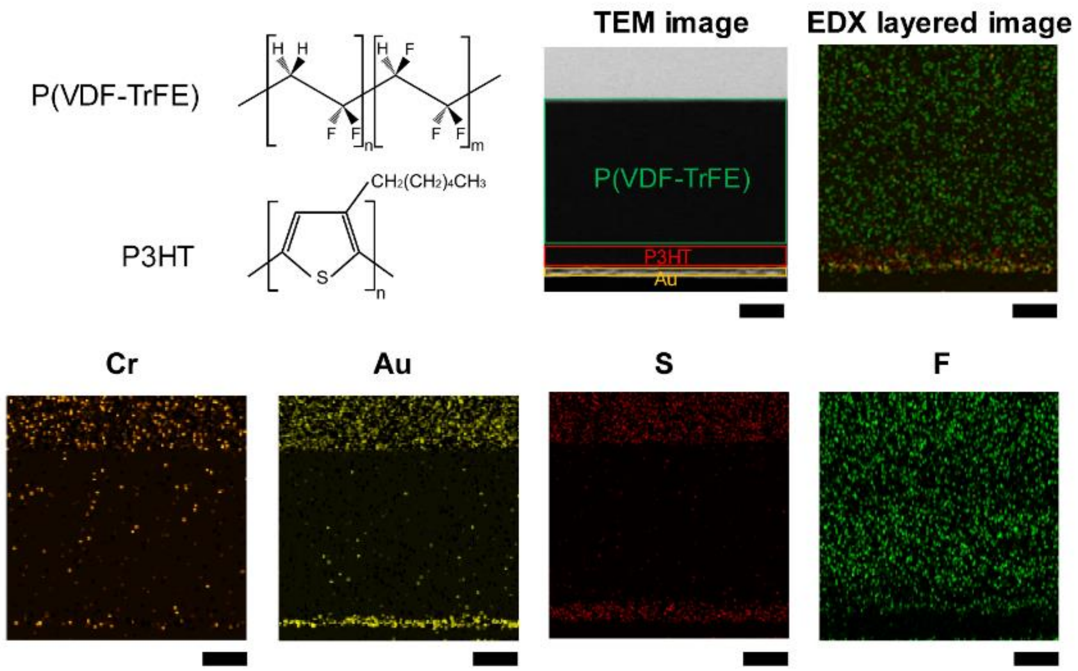

Figure S3. Cross-sectional TEM image and EDX mapping of ATFES. Scale bar: 200 nm.

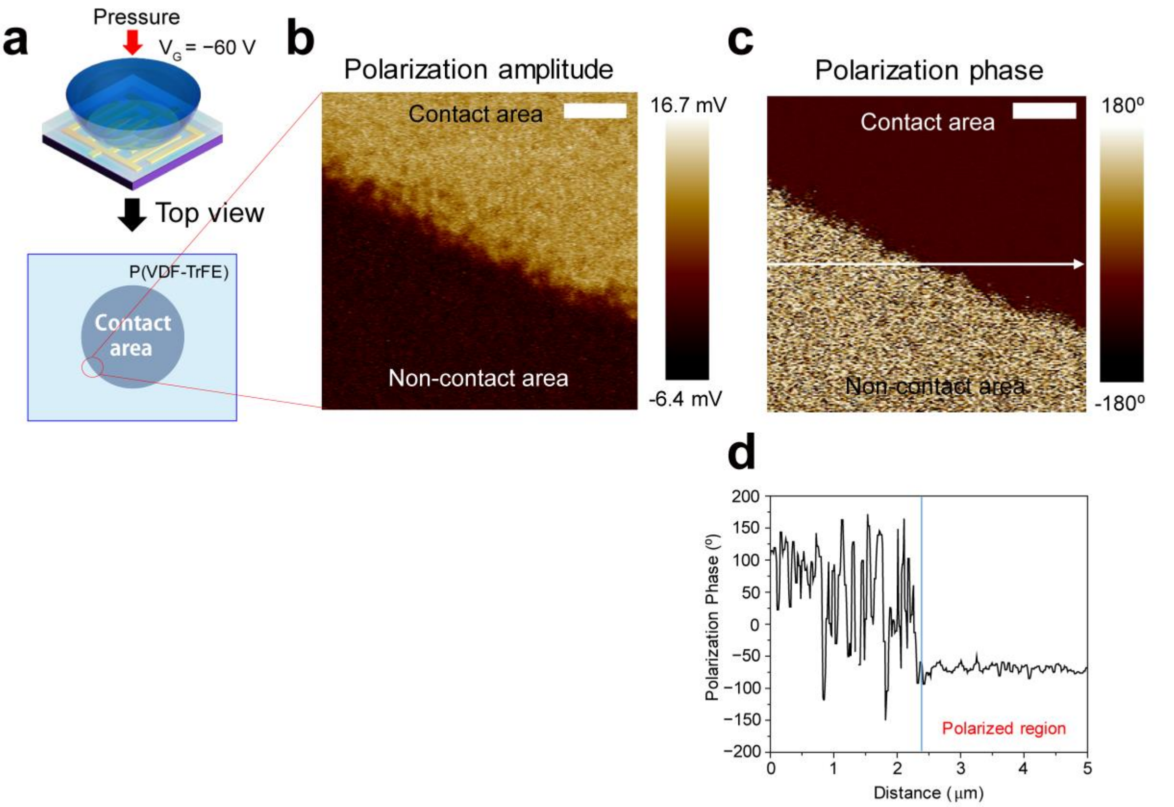

Figure S4. PFM images. (a) Schematic of the ATFES device and contact area between the gate electrode and P(VDF-TrFE) layer at a certain pressure. AFM images of (b) polarization amplitude, and (c) polarization phase at the boundary of the contact area at V_G = −60 V. (d) Polarization phase profile along the white line in (c). Scale bar: 1μm.

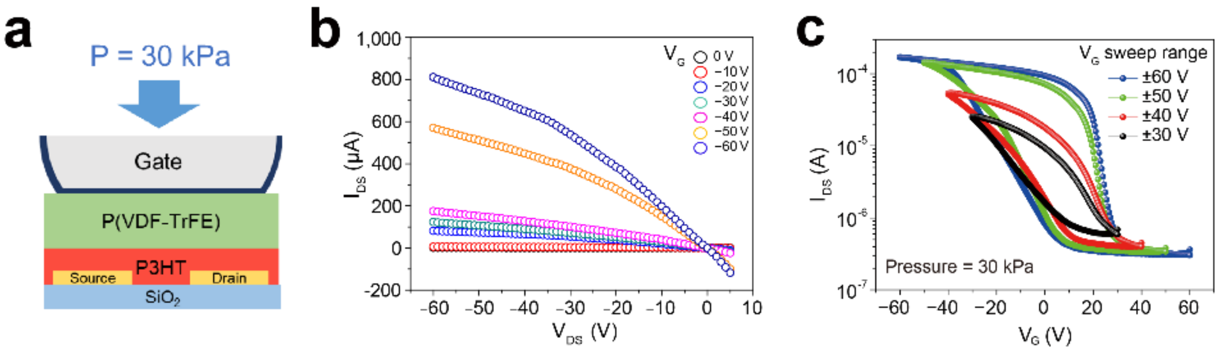

Figure S5. I_{DS}-V_{DS} output curve under various gate voltages and I_{DS}-V_G transfer curve at a fixed pressure of 30 kPa. (a) Schematic illustration of the ATFES device at 30 kPa. (b) Output curves of the device with V_G = 0 V, −10 V, −20 V, −30 V, −40 V, −50 V, and −60 V at a constant pressure of 30 kPa. (c) I_{DS}-V_G transfer curve with fixed input pressure (30 kPa) and different V_G ranges (V_G sweeping range = ±30 V, ±40 V, ±50 V, and ±60 V, respectively).

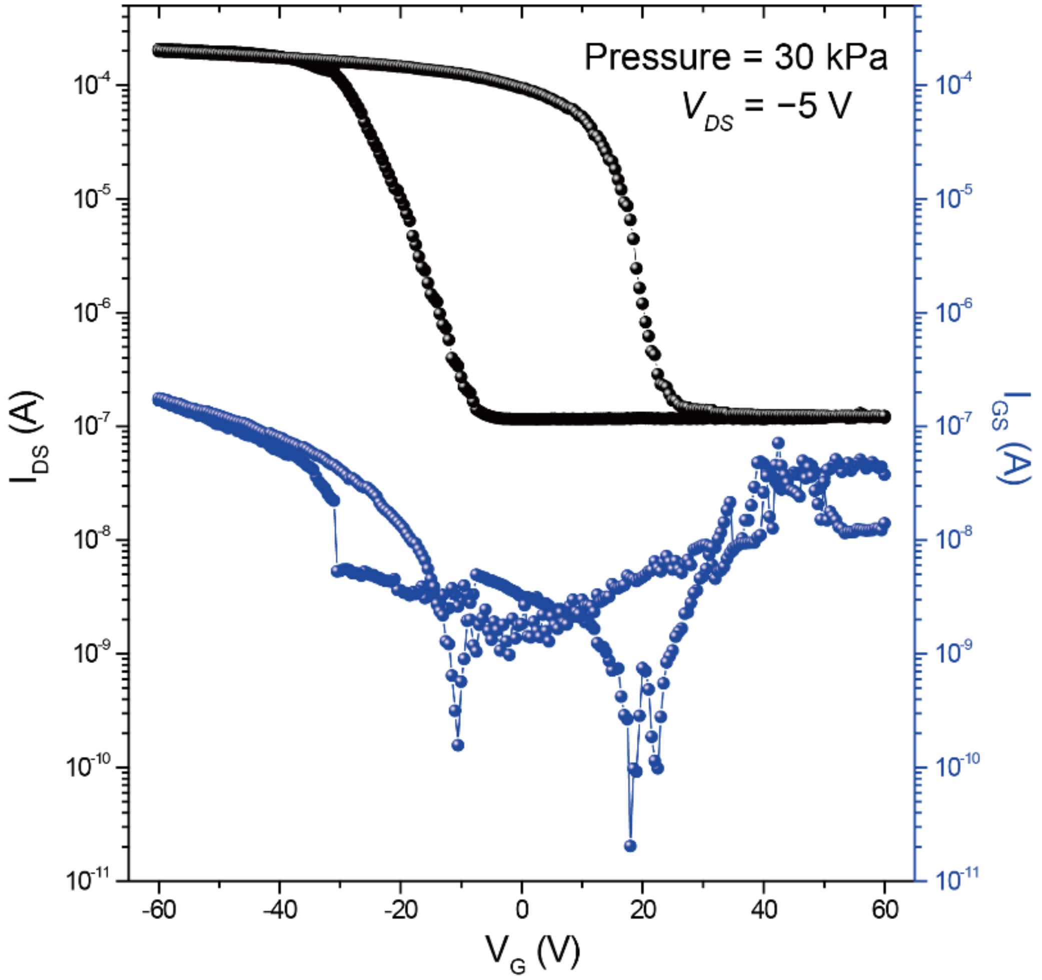

Figure S6. The I_{DS}-V_G and I_{GS}-V_G characteristics obtained under the V_G double sweep from –60 V to +60 V and V_{DS} = –5 V at a fixed pressure of 30 kPa. The gate current behavior with two distinct current drops was observed due to the polarization switching at the coercive voltage. The power consumption in programming process was about 1.01×10^{-5} W (V_G = –60 V and I_{GS} = -1.68×10^{-7} A) and in reading process, each power consumption for 'ON' and 'OFF' state was about 4.71×10^{-4} W (V_{DS} = –5 V and I_{DS} = -9.42×10^{-5} A) and 5.78×10^{-7} W (V_{DS} = –5 V and I_{DS} = -1.16×10^{-7} A), respectively.

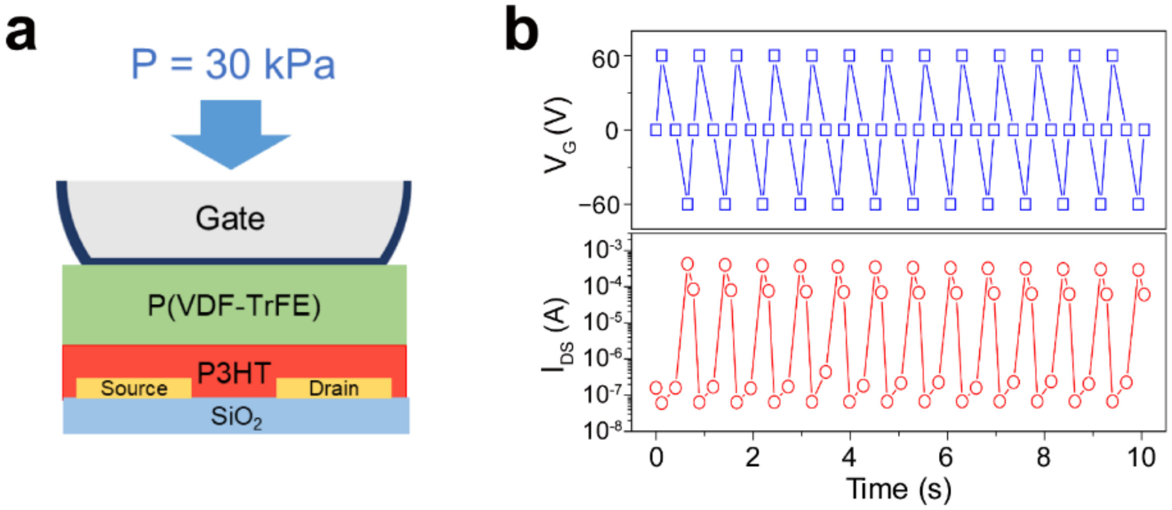

Figure S7. Dynamic switching response to consecutively changing gate voltage. (a) Schematic illustration of the ATFES device at 30 kPa. (b) Dynamic switching response of the device. 1 cycle: V_G = 0 V, +60 V, 0 V, and −60 V at constant pressure of 30 kPa. The program/erasure switching time was approximately 450 ms, comparable with previously reported results[14].

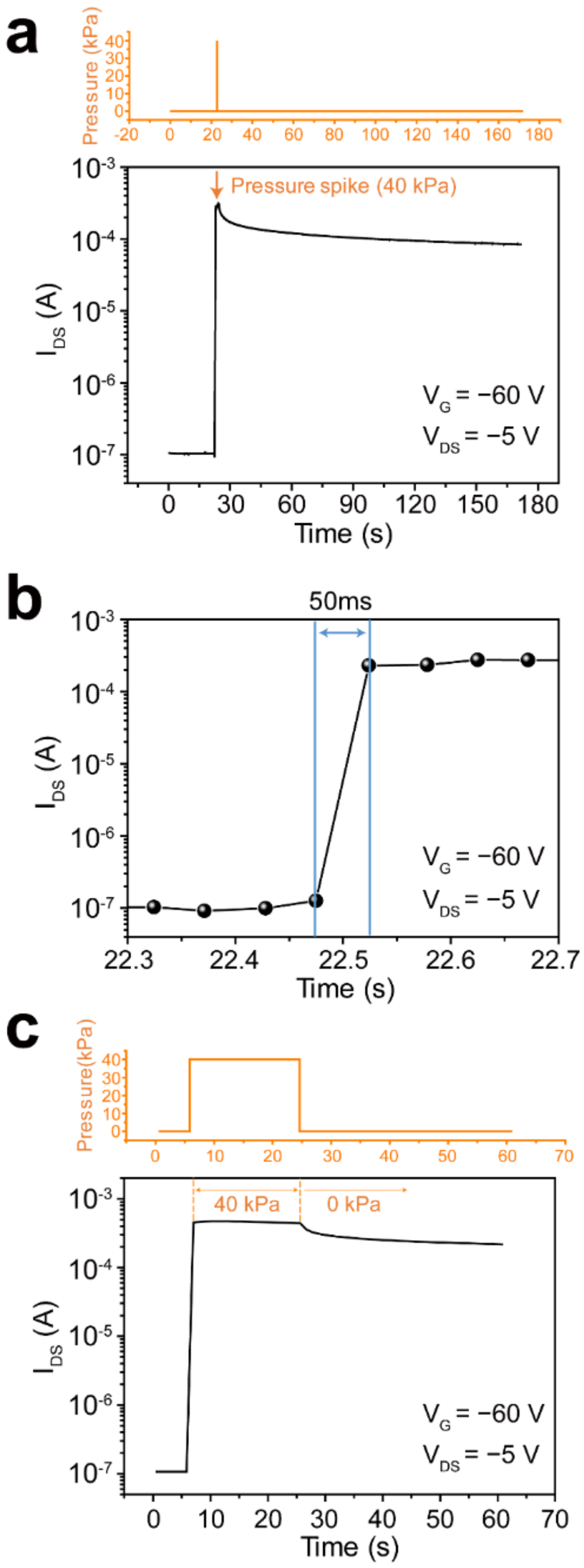

Figure S8. Real-time responses to pressure. (a) I_{DS} response to instant pressure (pressure-spike), (b) response time to pressure, (c) response to constant pressure (40 kPa) which is applied for a few seconds and then removed. No notable current drop was observed when the pressure was applied for longer than 5 s.

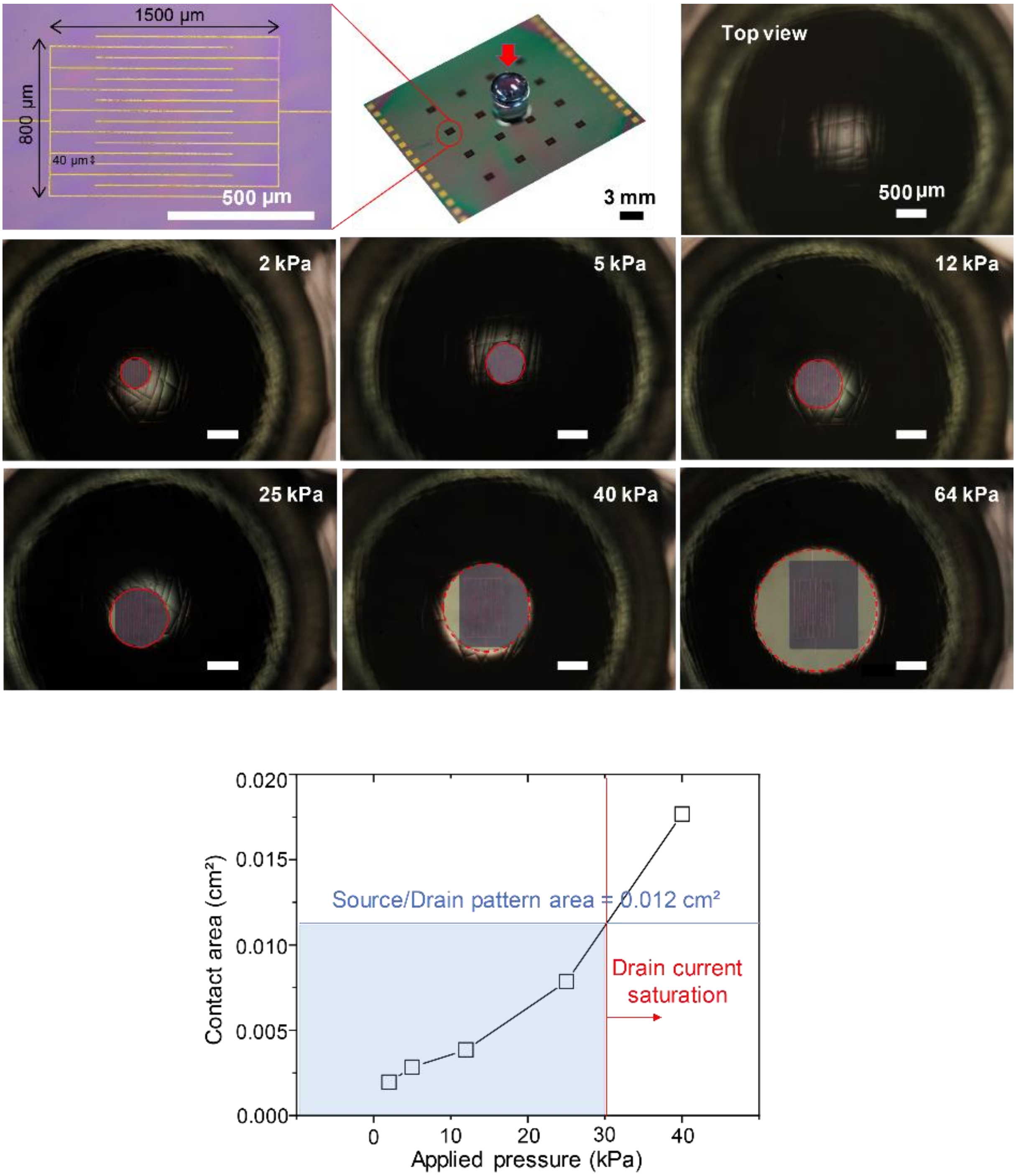

Figure S9. Optical microscope images for a top view as a function of pressure. The contact area is marked with red dotted lines. The circle shape—a section of the sphere—increases with increasing pressure. The graph below shows the contact area between the gate electrode and ferroelectric layer as a function of pressure.

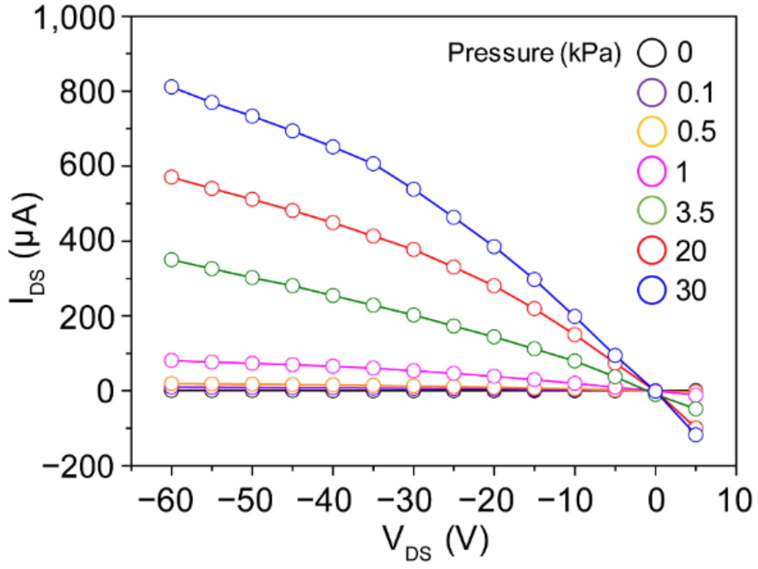

Figure S10. *I_{DS}*-*V_{DS}* output curve under various tactile pressures. Output curves with various pressures: 0 kPa, 0.1 kPa, 0.5 kPa, 1 kPa, 3.5 kPa, 20 kPa and 30 kPa at constant V_G = −60 V.

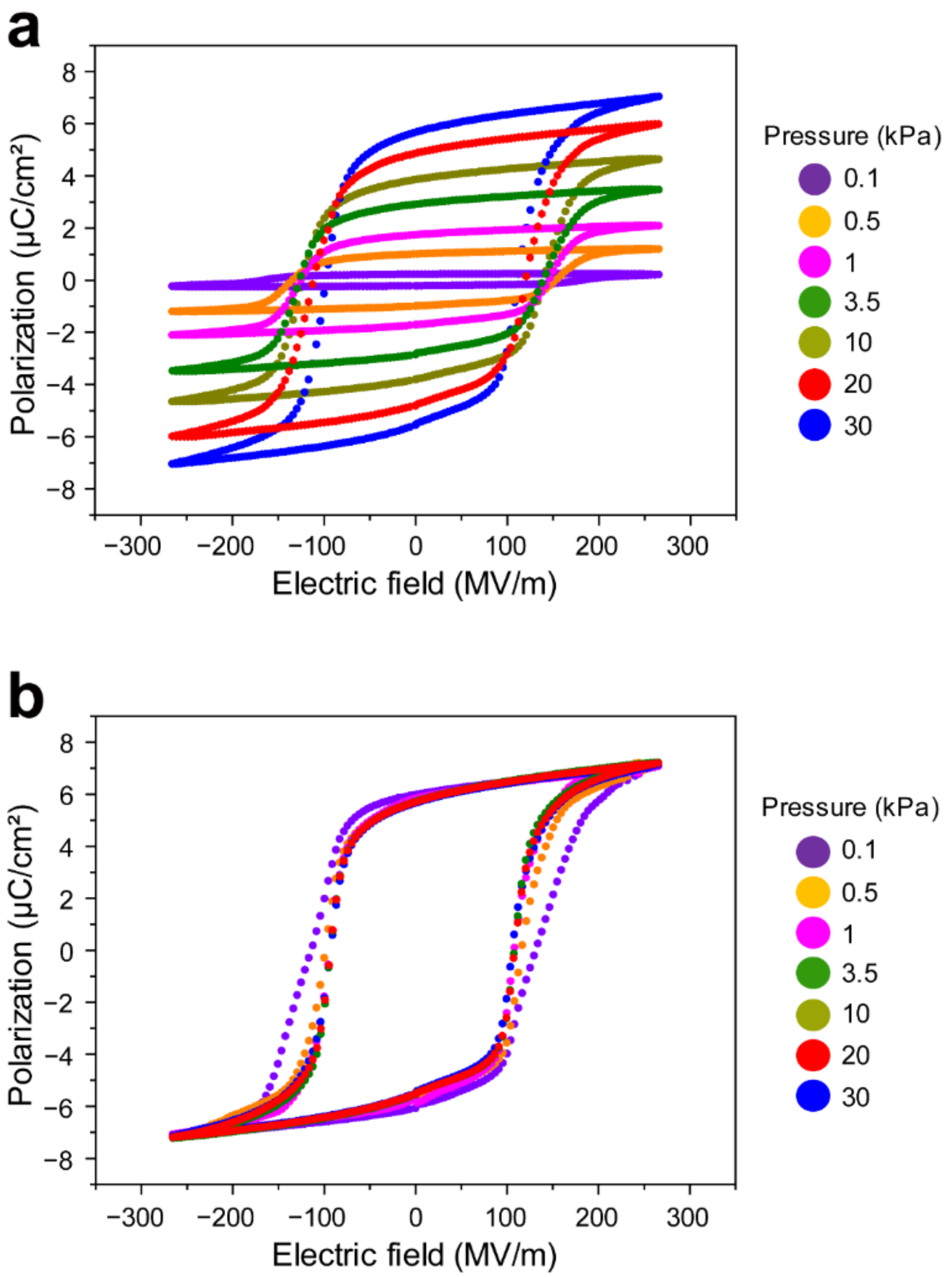

Figure S11. Polarization(P)–Electric field(E) curves of P(VDF-TrFE) layer in ATFES device. (a) Polarizations at various pressures (0.1 kPa, 0.5 kPa, 1 kPa, 3.5 kPa, 10 kPa, 20 kPa and 30 kPa) are calculated using the whole interdigitated source/drain area: 0.012 cm^2. (b) Polarizations at various pressures (0.1 kPa, 0.5 kPa, 1 kPa, 3.5 kPa, 10 kPa, 20 kPa and 30 kPa) are calculated using the contact area of each pressure.

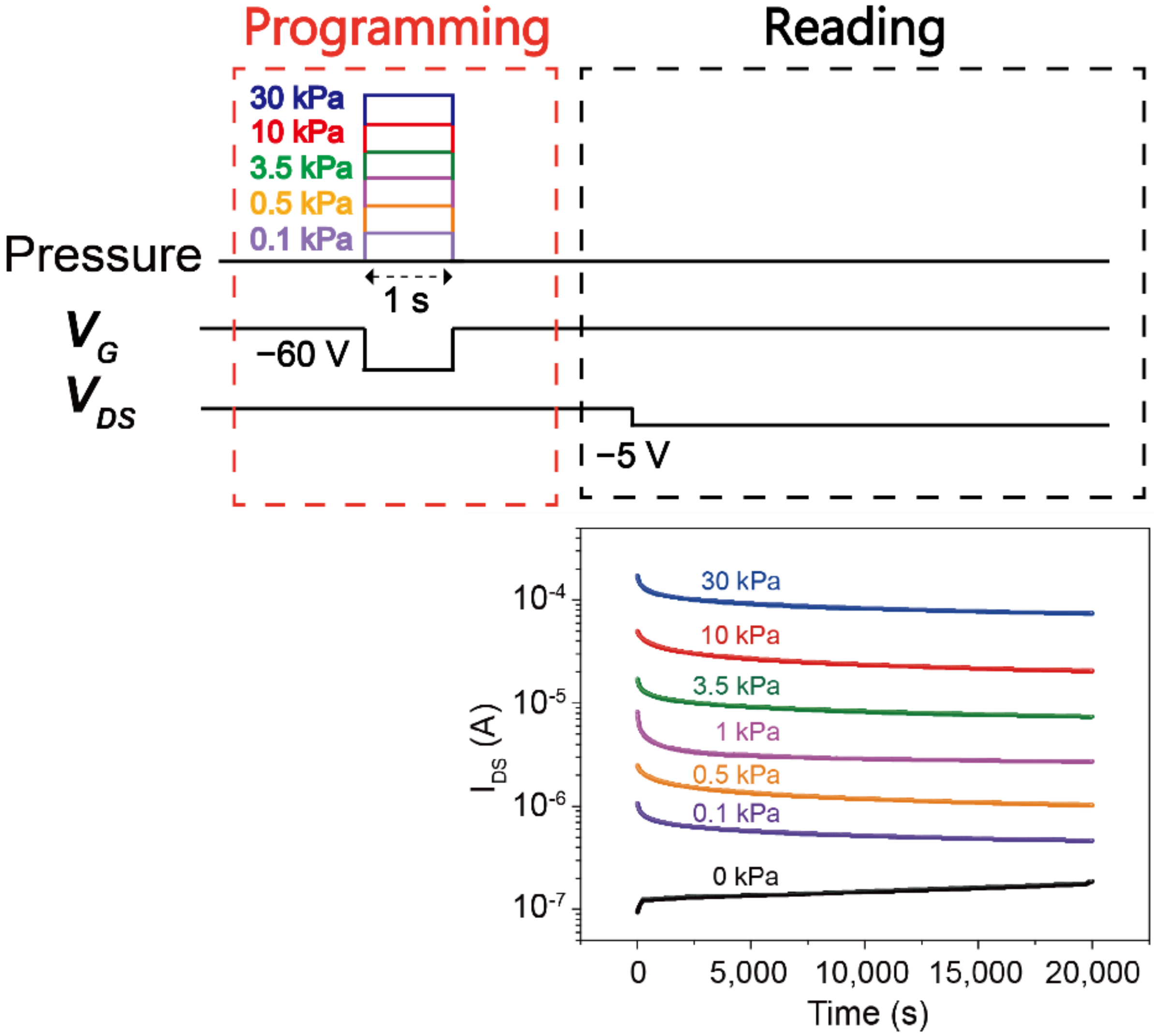

Figure S12. The measuring sequence of I_{DS} retention characteristics with different pressures. The pressures ranging from 0 to 30 kPa and voltage of $V_G = -60$ V were applied for 1 s for programming process. In reading process, $V_G = 0$ V, $V_{DS} = -5$ V were applied up to 20,000 s.

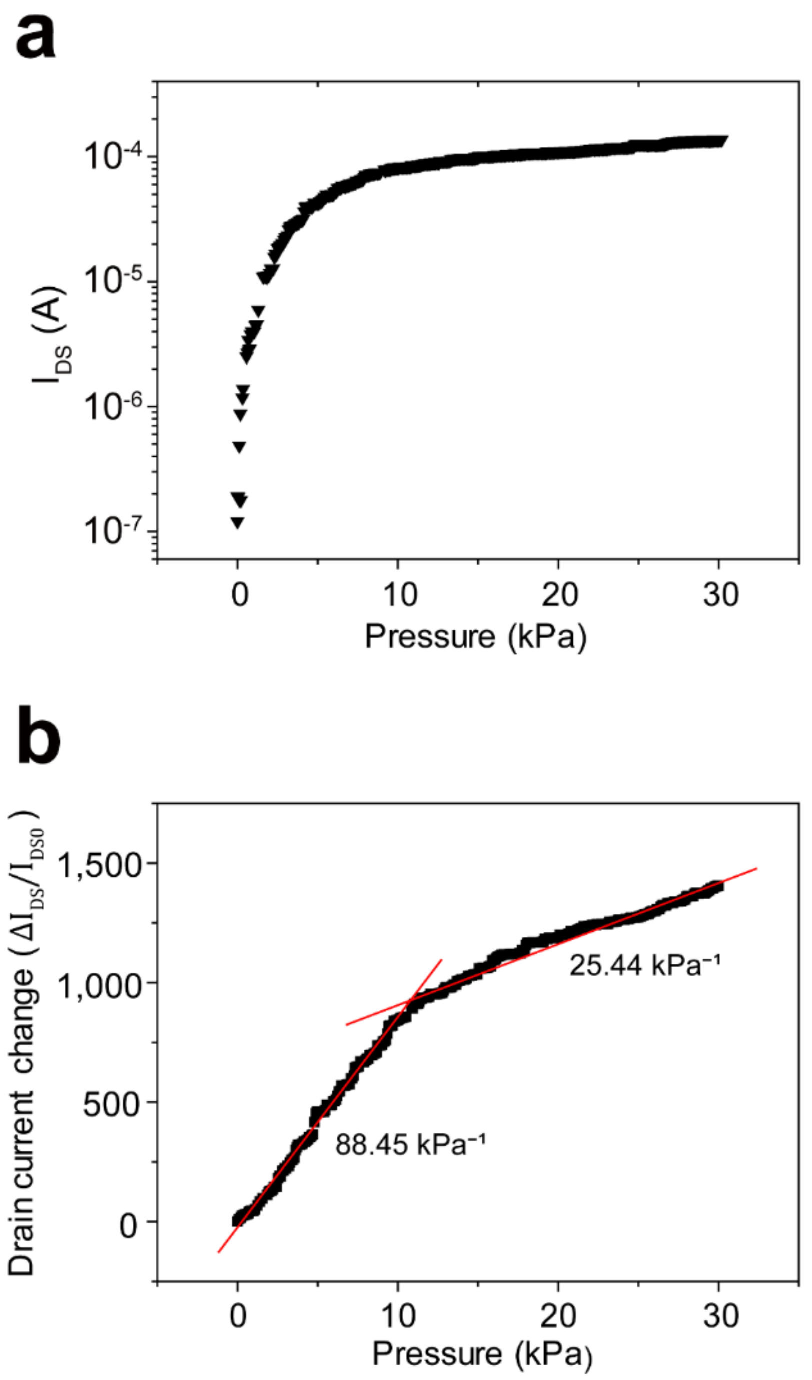

Figure S13. I_{DS} as a function of pressure and pressure sensitivity of the ATFES device under V_G = −60 V. (a) I_{DS} as a function of applied pressure. (b) Relative drain current change as a function of applied tactile pressure**.** The pressure sensitivity (S_P) is defined as $S_P = \delta(\Delta I_{DS} / I_{DS0})/\delta p$, where p is the applied tactile pressure on the gate electrode, and I_{DS} and I_{DS0} are the drain-source current with and without pressure, respectively.

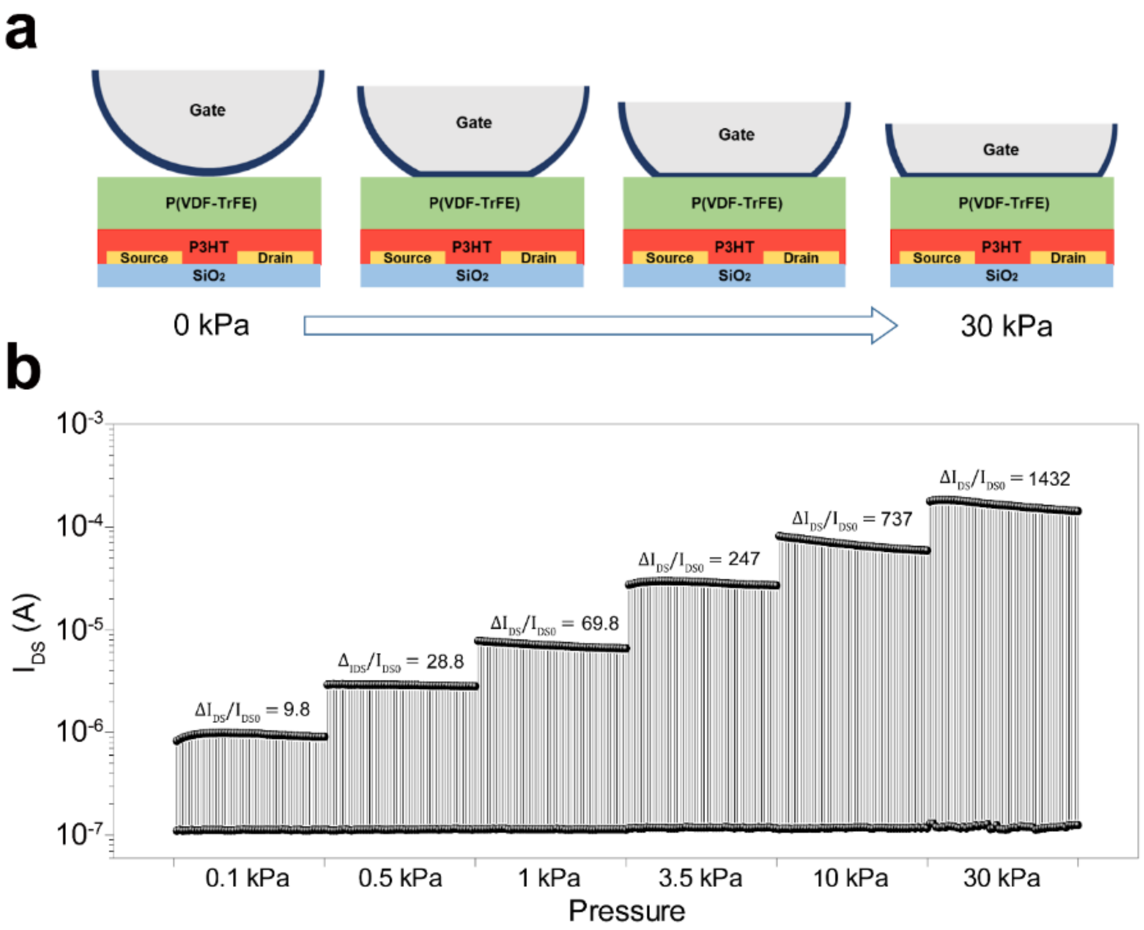

Figure S14. Program/erase cycle endurance test under various pressures. (a) Schematic illustrations of ATFES device from 0 kPa to 30 kPa. (b) Memory endurance test with 50 cycles for each pressure. 1 cycle consists of V_G = 0 V, +60 V, 0 V, and −60 V. Each distinct level of ON and OFF I_{DS} was examined at V_G = 0 V and V_{DS} = −5 V after programmed and erased at V_G = −60 V and V_G = +60 V, respectively.

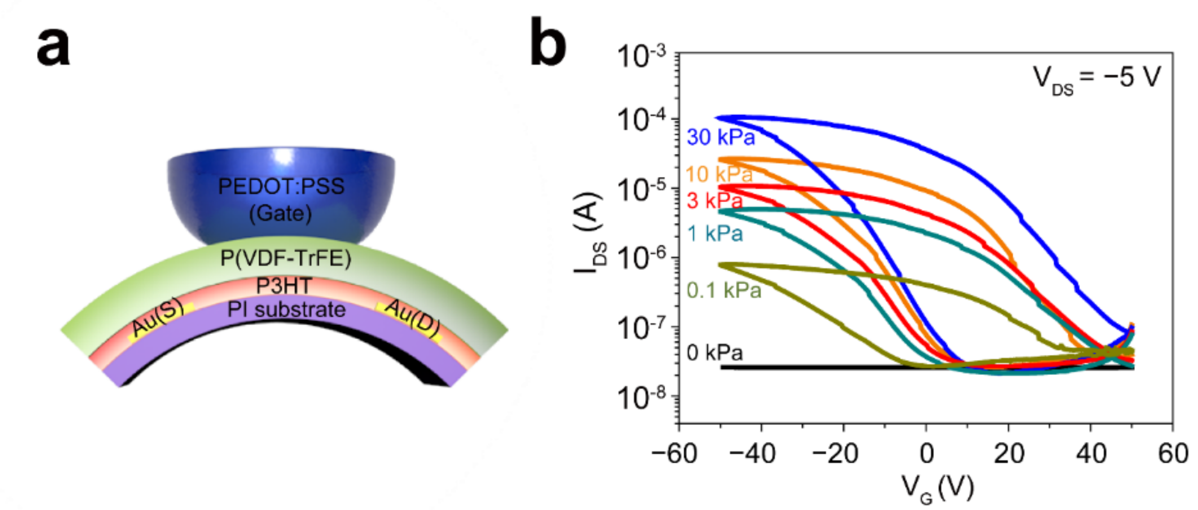

Figure S15. I_{DS}-V_G transfer curve of flexible ATFES at different pressures. (a) Schematic illustration of ATFES device on a flexible substrate (polyimide (PI) substrate). (b) Transfer curves at different pressures: 0 kPa, 0.1 kPa, 1 kPa, 3 kPa, 10 kPa, 30 kPa. V_{DS} = −5 V.

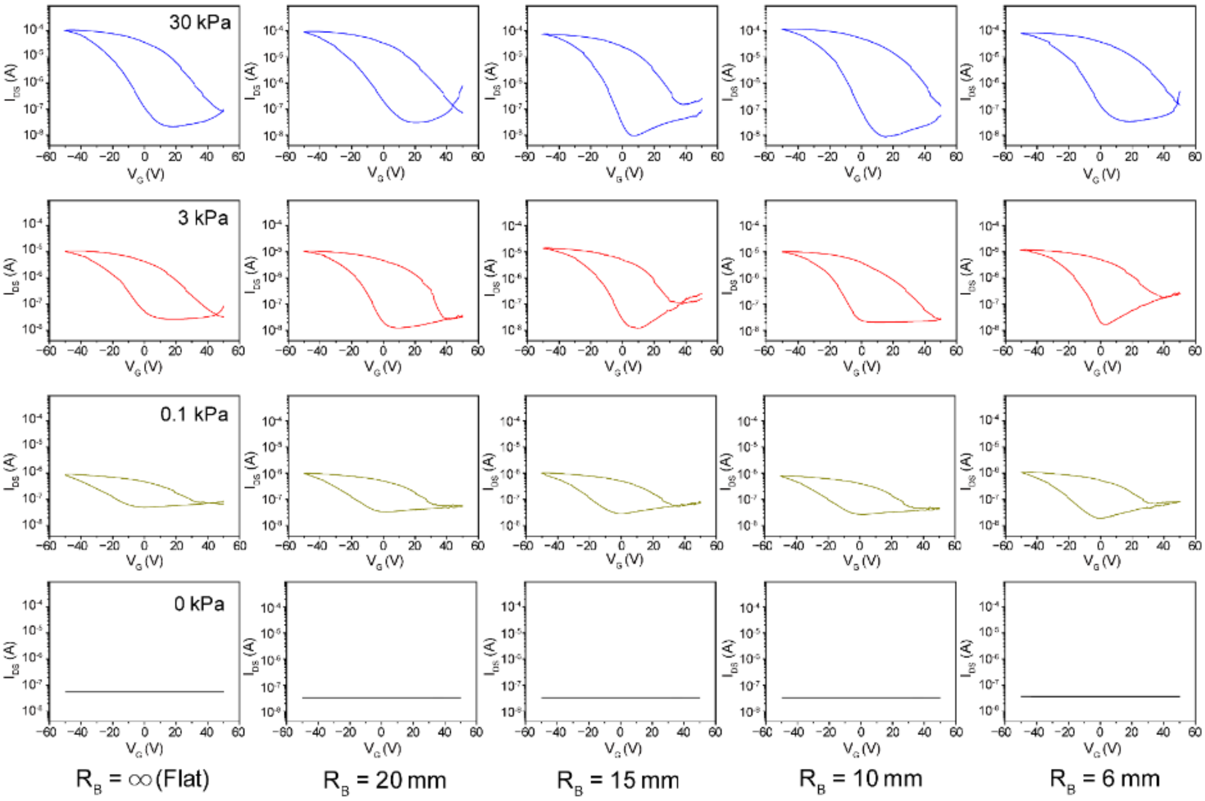

Figure S16. I_{DS}-V_G transfer curve of flexible ATFES under various bending radii. I_{DS}-V_G hysteresis curves programmed with different pressure: 0 kPa, 0.1 kPa, 3 kPa, and 30 kPa at a specific bending radius (R_B = ∞(flat), 20, 15, 10, 6 mm). R_B indicates the bending radius.

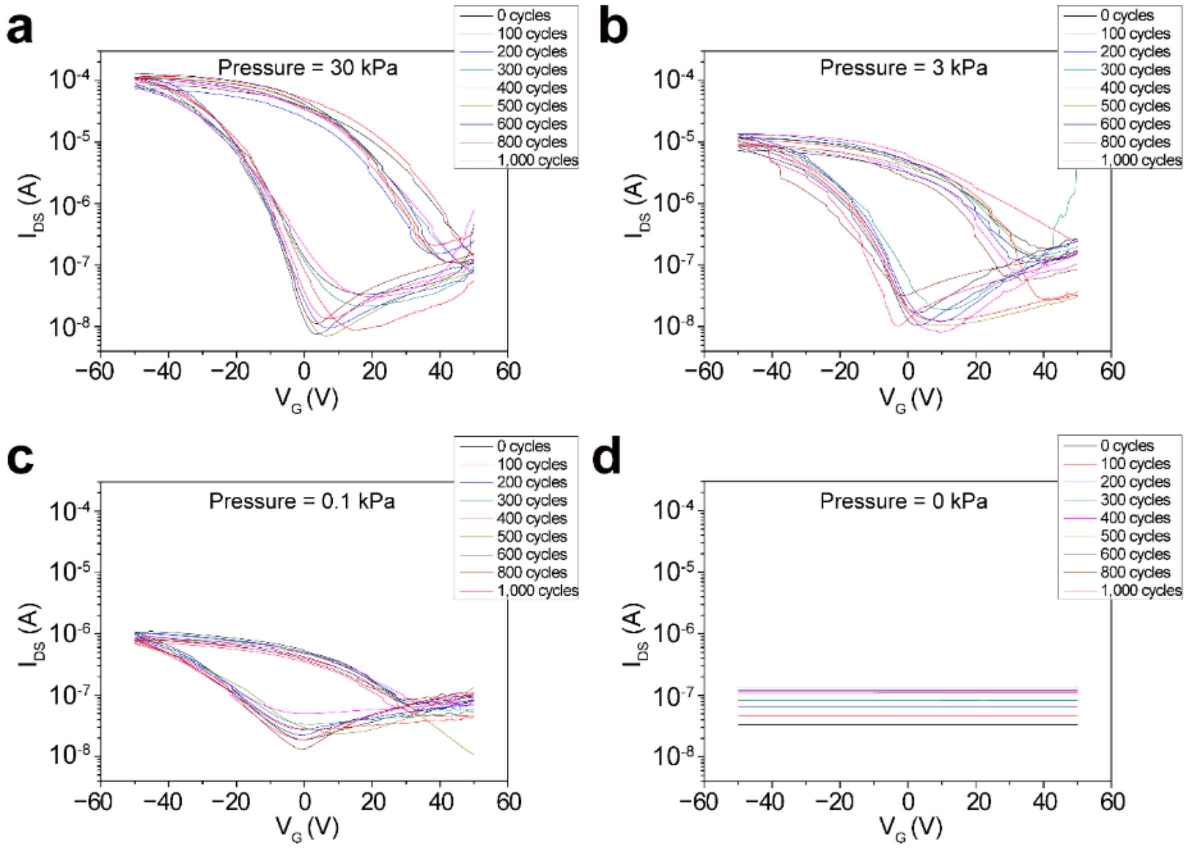

Figure S17. ***I_{DS}-V_G* transfer curve of flexible ATFES under various bending cycles.** *I_{DS}-V_G* hysteresis curves after specific bending cycles programmed with different pressures: (a) 30 kPa, (b) 3 kPa, (c) 0.1 kPa, (d) 0 kPa.

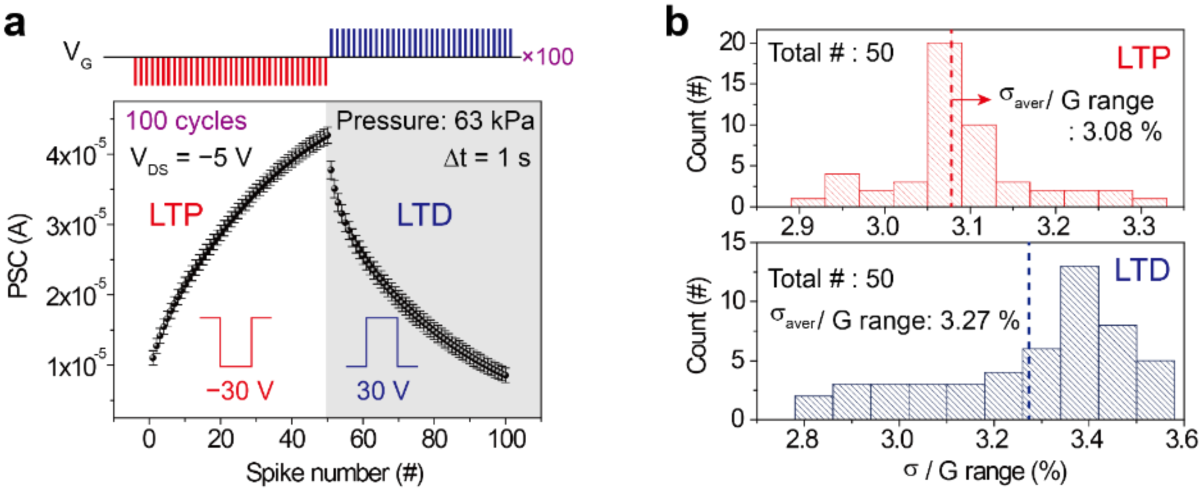

Figure S18. Statistical analysis of repetitive LTP and LTD with continuous input pulses. (a) The statistical LTP and LTD transition during 100 cycles with input pulses of V_G = ±30 V for 500 ms and V_{DS} = −5 V at ~63 kPa. (b) Histogram of the standard deviation (σ) of the conductance (*G*) for each interstate in LTP/LTD. For each average value of σ for LTP/LTD is divided by *G* transition range, and defined as the variability (σ_{aver}/*G range*), resulting in 3.08 %, 3.27 % respectively. The total σ_{aver}/*G range* in LTP and LTD transition was 3.18 %.

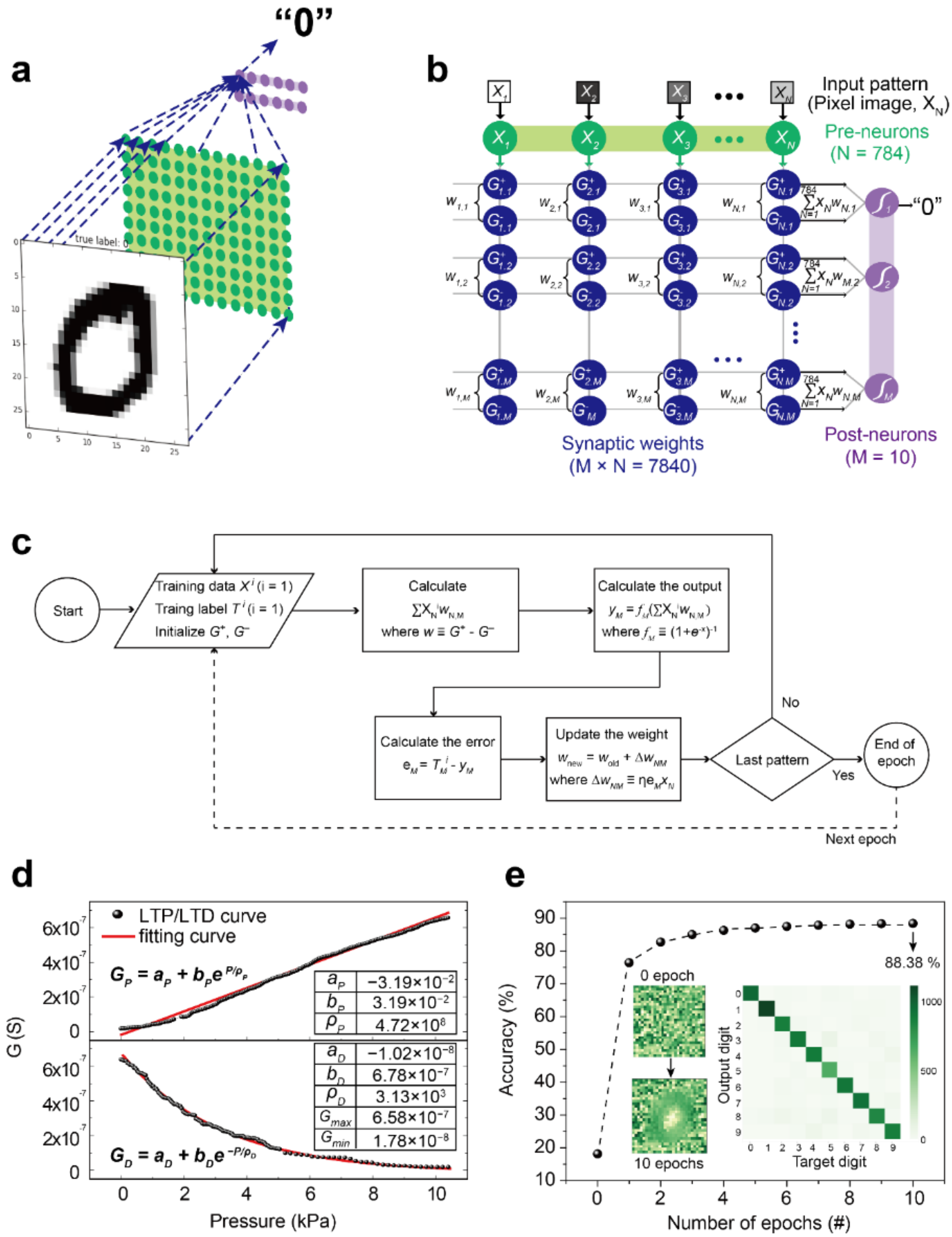

Figure S19. MNIST pattern recognition simulation and learning algorithm. (a) Constituents of a single-layer neural network for the MNIST pattern recognition. (b) Array diagram mapped to the single-layer neural network. (c) Flowchart of the training process based on the backpropagation algorithm. (d) Analog potentiation (upper panel) and depression (lower panel) of conductance (G) with respect to the applied pressure at V_G of ±30 V. The solid lines are the best fit results for $G = a_{P(D)} + b_{P(D)}exp(+(-)P/\rho_{P(D)})$ for LTP (LTD) curve, where $a_{P(D)}$, $b_{P(D)}$, and $\rho_{P(D)}$ are fitting parameters (inset table). (e) The recognition accuracy for MNIST patterns as a function of epoch number. The left insets show a reshaped 28 × 28 contour image of the digit "0" from weight (w) before and after 10 epochs. The right inset is

the confusion matrix for a recognition test after learning 10 epochs. A recognition accuracy of 88.38 % was achieved after 10 epochs.

The MNIST pattern recognition simulation method

Pattern recognition simulation was performed by using the MNIST digits dataset consisting of 60,000 training images and 10,000 test images.[15] A single-layer neural network for the MNIST pattern recognition is composed of 784 pre-neurons, 10 post-neurons, and 7,840 synaptic weights, as shown in a Figures S19a and b. In this network, the 784 input (X_N, $N = 1,2,\ldots, 784$) pixels of the MNIST digit pattern are connected to the pre-neurons in order. Each output (y_M, $M = 1,2,\ldots, 10$) is determined by a sigmoid activation function ($f_M = (1 + e^{-x})^{-1}$), where x is a result of the matrix-vector multiplication using X_N and the corresponding synaptic weight ($w_{NM} = G^{+}_{NM} - G^{-}_{NM}$). Note that the subscripts N and M correspond to the ordinal numbers of pre- and post-neurons. As shown in the flowchart of Figure S19c, it is set that all G^{+} and G^{-} are randomly initialized in the ranged of G_{max} and G_{min}. And the training datasets (X^i, i = 1, 2, …, 60,000) are fed to the single-layer network. Each X^i has its own training label (T^i) denoting an integer ranged from 0 to 9. Since $y_M = f_M(\sum X^i_N w_{NM})$), the error value ($e_M$) = $T^i - y_M$. Based on the delta rule algorithm ($\Delta w_{NM} = \eta e_M X_N$, where η is a learning rate), the old weights (w_{old}) are updated to new weights ($w_{new} = w_{old} - \Delta w_{NM}$). When 60,000 training data goes through this process, one epoch is completed. The fitting results of conductance change for LTP (upper panel) and LTD (lower panel) with respect to applied pressure are shown in Figure S19d, when V_G =

30 V(−30 V) is applied for LTP (LTD), respectively. And each LTP/LTD curve is fitted based on the following equation, respectively;

$$G_P = a_P + b_P e^{P/\rho_P} \text{ and } G_D = a_D + b_D e^{-P/\rho_D}$$

where G_P and G_D represent the conductance for LTP and LTD, respectively; a_P, b_P, ρ_P, a_D, b_D, and ρ_D are the fitting parameters; and the subscripts P and D denote potentiation and depression, respectively (inset table of Figure S19d). Since experimental $G_{P(D)}$ that has a limited range from G_{min} to G_{max} reflect the device own performance, the total amount of Δw can be constrained. With this recognition process based on the fitting parameters, we achieved 88.38 % recognition accuracy after 10 epochs, as shown in Figure S19e. We should note here that same method and fitting parameters are used for recognizing the handwriting patterns, as shown in Figure 4e. The more details of the learning algorithm are described in MNIST pattern recognition simulation section of a previously reported article.[16]

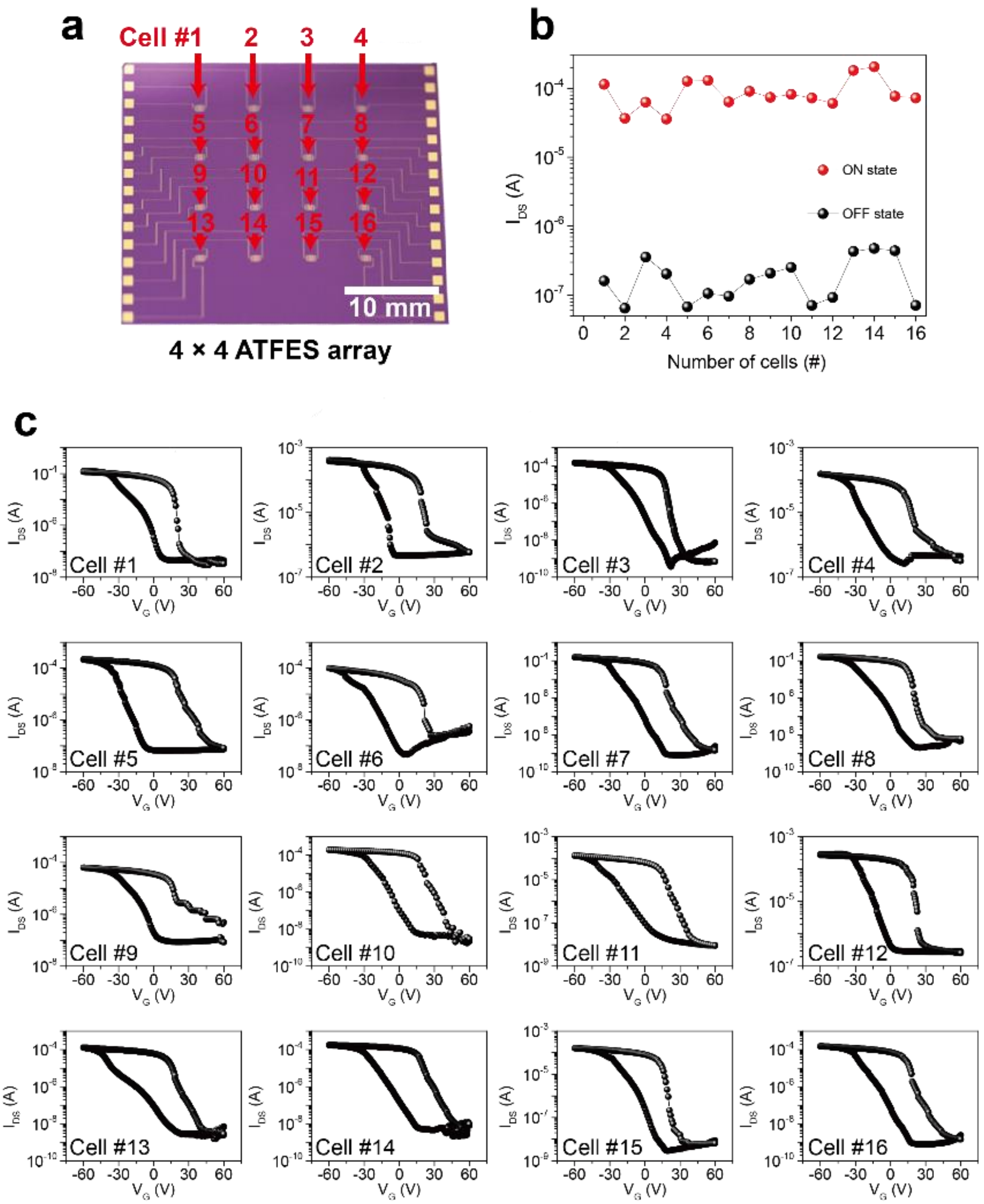

Figure S20. I_{DS}-V_G transfer characteristics of 16 cells of the 4 × 4 ATFES array. (a) Optical microscope image of the 4 × 4 ATFES array. (b) Statistical plot of I_{DS} for 'ON' and 'OFF' states of each cell in the array. (c) I_{DS}-V_G curves of the 16 cells obtained during V_G double sweep ranging from +60 V to –60 V at V_{DS} = – 5V.

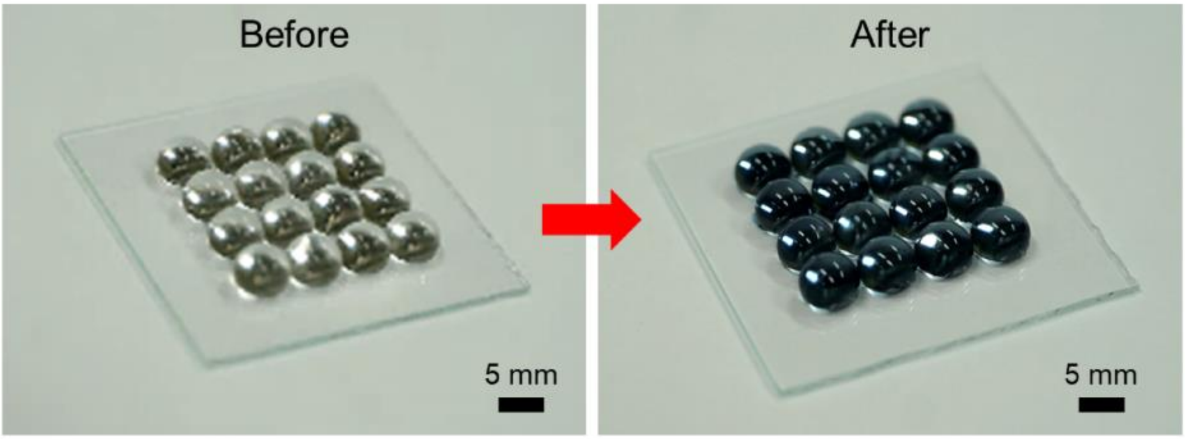

Figure S21. Photograph of arrays of PDMS hemispheres before and after being coated with PEDOT:PSS.

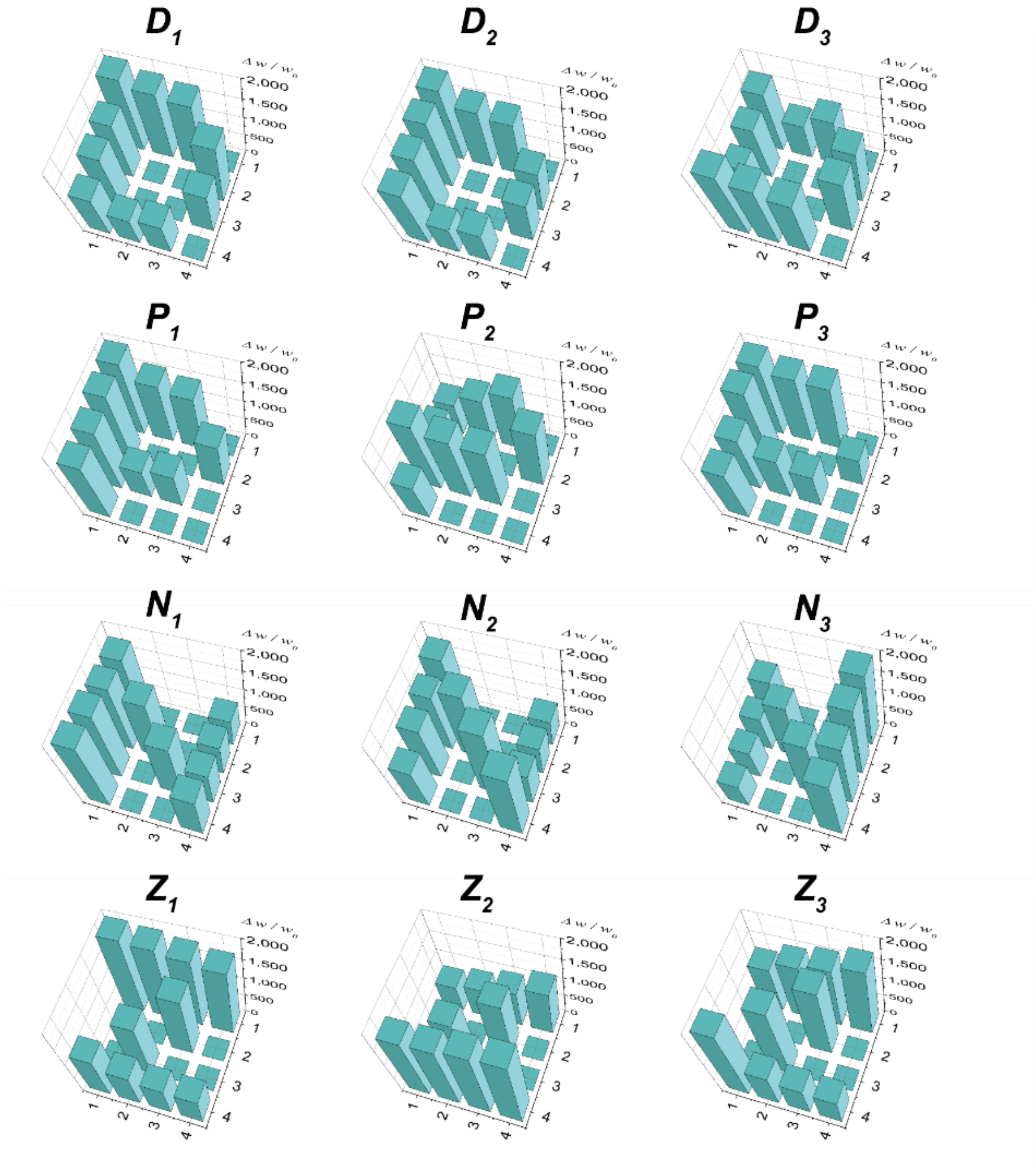

Figure S22. The histogram of $\Delta w/w_o$ in the 4 × 4 ATFES array for 12 alphabet character set. After encoding with the different magnitude of pressure-spike at V_G = –30 V, each PSC response for corresponding handwriting pattern is measured at V_{DS} = –5 V and V_G = 0 V on each node of the array, which is utilized in learning for handwriting pattern recognition.

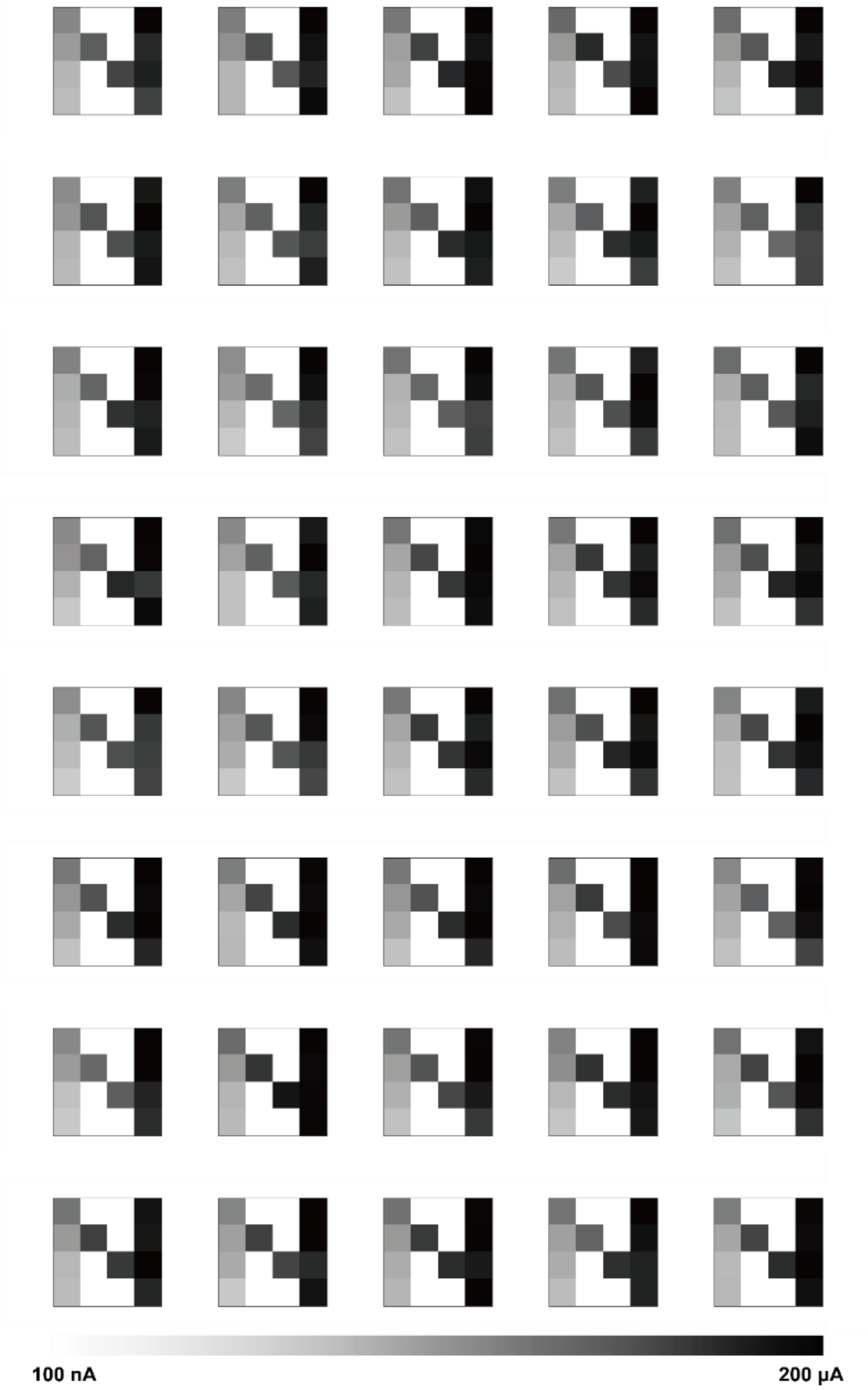

Figure S23. 40 examples of "N_3" pattern dataset generated with NF value of 10 %. For each pattern, 100 images were generated, resulting in a total of 1200 datasets. Following this, 900 pattern data were used for training, and 300 for testing.

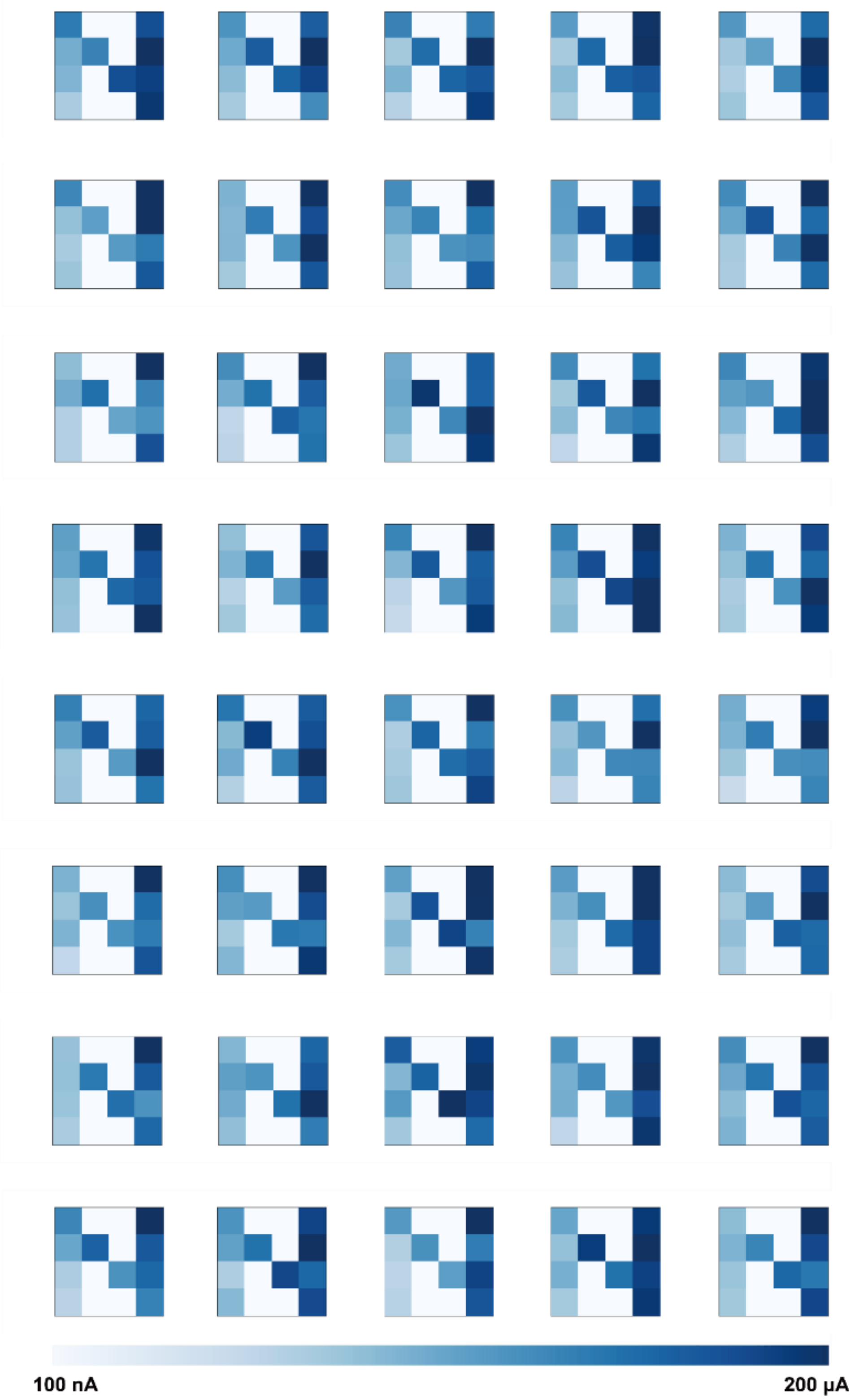

Figure S24. 40 examples of “N_3” pattern dataset generated with NF value of 25 %.

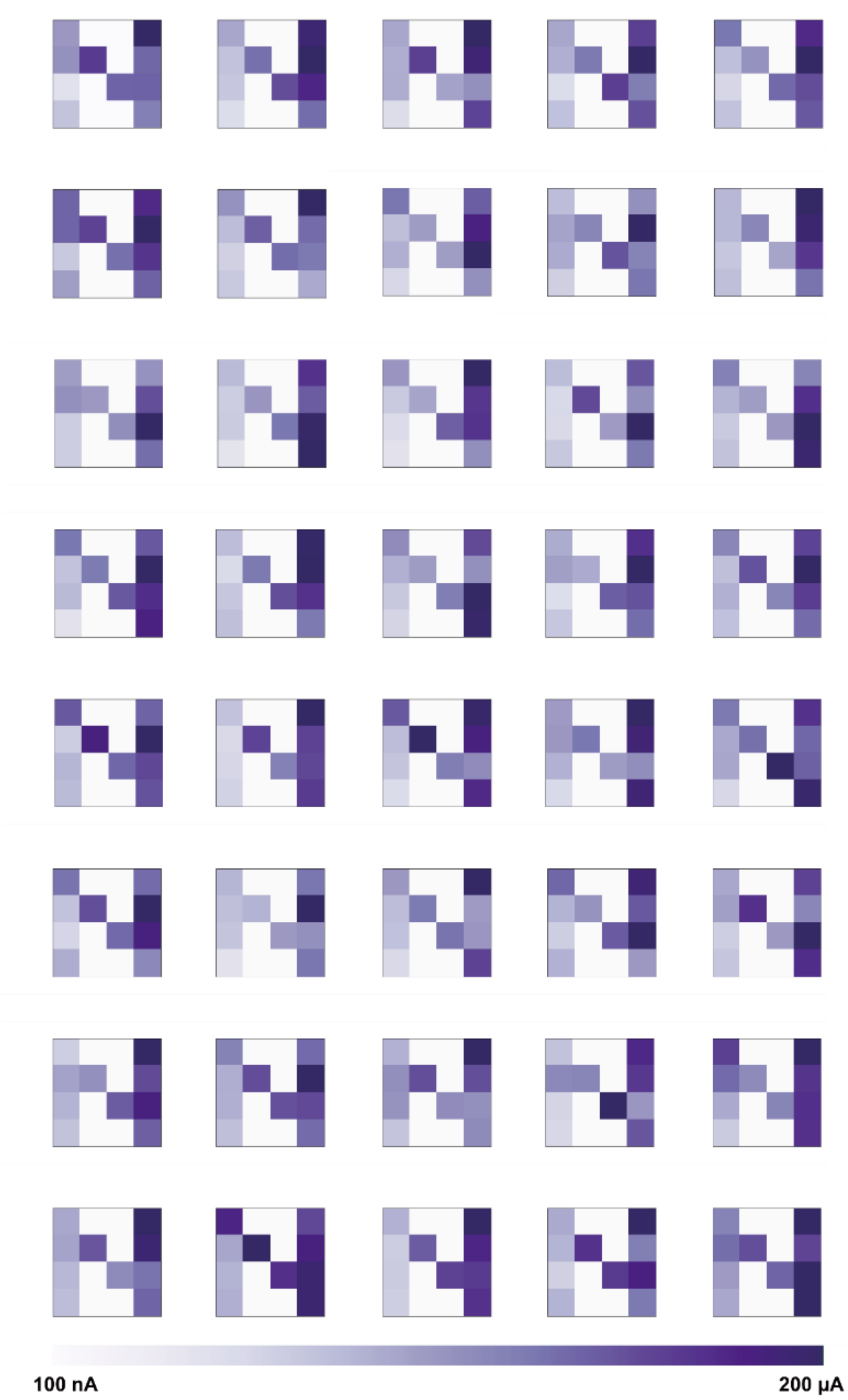

Figure S25. 40 examples of "N_3" pattern dataset generated with NF value of 40 %.

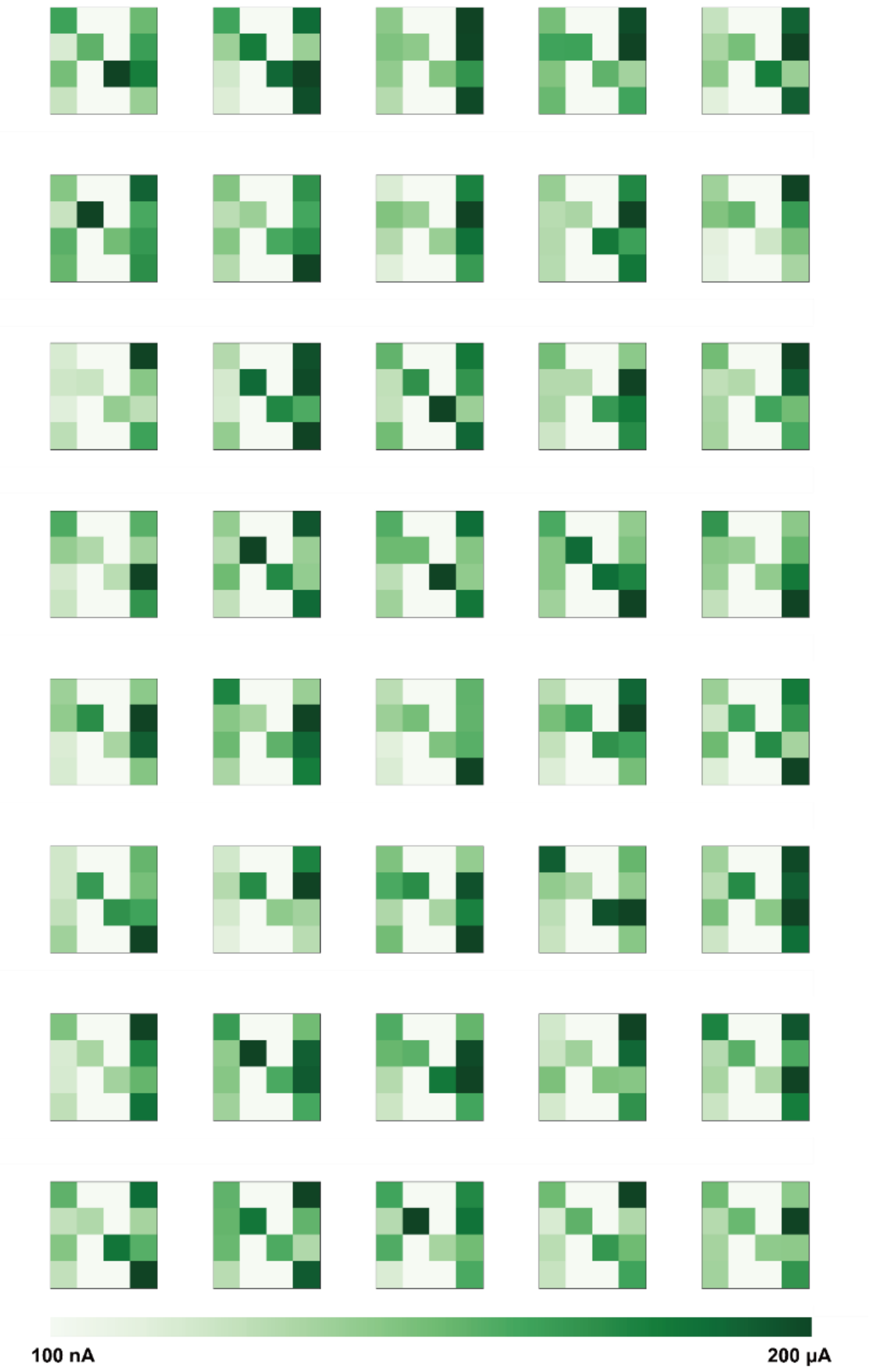

Figure S26. 40 examples of "N_3" pattern dataset generated with NF value of 60 %.

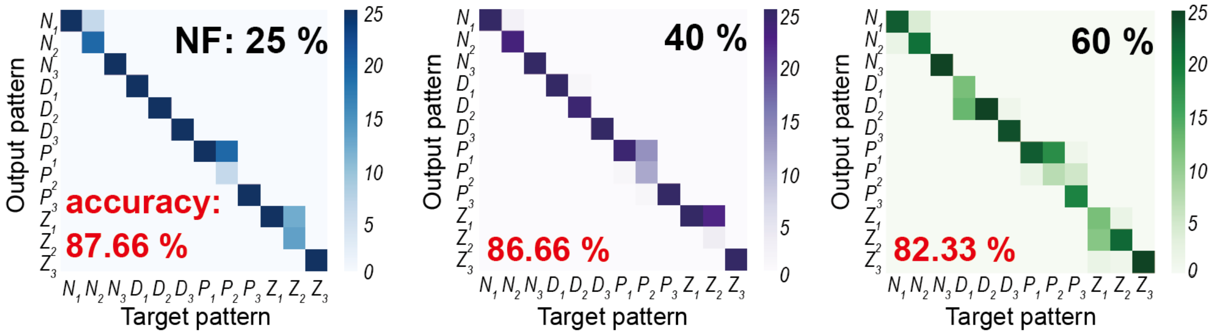

Figure S27. Confusion matrices between output and target patterns for a classification test of four alphabet characters ("*D*," "*P*," "*N*," "*Z*") with three different handwriting styles (1, 2, 3) with respect to different NF values (NF : 25 %, 40 %, and 60 %).

[1] T. Sekitani, T. Yokota, U. Zschieschang, H. Klauk, S. Bauer, K. Takeuchi, M. Takamiya, T. Sakurai, T. Someya, *Science* **2009**, *326,* 1516.

[2] G. Casula, P. Cosseddu, A. Bonfiglio, *Adv. Electron. Mater.* **2015**, *1*, 1500234.

[3] B. Zhu, H. Wang, Y. Liu, D. Qi, Z. Liu, H. Wang, J. Yu, M. Sherburne, Z. Wang, X. Chen, *Adv. Mater.* **2016**, *28*, 1559.

[4] K. Kanao, T. Arie, S. Akita, K. Takei, *J. Mater. Chem. C* **2016**, *4*, 9261.

[5] Y. Sun, X. Zheng, X. Yan, Q. Liao, S. Liu, G. Zhang, Y. Li, Y. Zhang, *ACS Appl. Mater. Interfaces* **2017**, *9*, 43822.

[6] K. Kanao, S. Nakata, T. Arie, S. Akita, K. Takei, *Mater. Horiz.* **2017**, *4*, 1079.

[7] C. T. Chen, W. Y. Lee, T. L. Shen, H. C. Wu, C. C. Shih, B. W. Ye, T. Y. Lin, W. C. Chen, Y. F. Chen, *Adv. Electron. Mater.* **2017**, *3*, 1600548

[8] Y. Zang, H. Shen, D. Huang, C. A. Di, D. Zhu, *Adv. Mater.* **2017**, *29,* 1606088

[9] C. Wan, G. Chen, Y. Fu, M. Wang, N. Matsuhisa, S. Pan, L. Pan, H. Yang, Q. Wan, L. Zhu, *Adv. Mater.* **2018**, *30*, 1801291.

[10] C. Zhang, W. B. Ye, K. Zhou, H. Y. Chen, J. Q. Yang, G. Ding, X. Chen, Y. Zhou, L. Zhou, F. Li, *Adv. Funct. Mater.* **2019**, *29*, 1808783.

[11] X. Zhang, Y. Zhuo, Q. Luo, Z. Wu, R. Midya, Z. Wang, W. Song, R. Wang, N. K. Upadhyay, Y. Fang, *Nat. Commun.* **2020**, *11,* 1.

[12] Q. Sun, D. H. Ho, Y. Choi, C. Pan, D. H. Kim, Z. L. Wang, J. H. Cho, *ACS Nano* **2016**, *10,* 11037.

[13] Y. Liu, Z. Liu, B. Zhu, J. Yu, K. He, W. R. Leow, M. Wang, B. K. Chandran, D. Qi, H. Wang, *Adv. Mater.* **2017**, *29*, 1701780.

[14] K. L. Kim, W. Lee, S. K. Hwang, S. H. Joo, S. M. Cho, G. Song, S. H. Cho, B. Jeong, I. Hwang, J. H. Ahn, *Nano Lett.* **2016**, *16*, 334.

[15] D. Li. *IEEE Signal Process. Mag.* **2012**, 29, 141

[16] S. Choi, S. Jang, J.-H. Moon, J. C. Kim, H. Y. Jeong, P. Jang, K.-J. Lee, G. Wang, *NPG Asia Mater.* **2018**, 10, 1097.
